# Realistic modeling of mesoscopic ephaptic coupling in the human brain

**DOI:** 10.1371/journal.pcbi.1007923

**Published:** 2020-06-01

**Authors:** Giulio Ruffini, Ricardo Salvador, Ehsan Tadayon, Roser Sanchez-Todo, Alvaro Pascual-Leone, Emiliano Santarnecchi

**Affiliations:** 1 Neuroelectrics Corporation, Cambridge, Massachusetts, United States of America; 2 Neuroelectrics Barcelona, Barcelona, Spain; 3 Starlab Barcelona, Barcelona, Spain; 4 Berenson-Allen Center for Noninvasive Brain Stimulation, Beth Israel Deaconess Medical Center and Harvard Medical School, Boston, Massachusetts, United States of America; 5 Hinda and Arthur Marcus Institute for Aging Research and Center for Memory Health, Hebrew SeniorLife, Boston, Massachusetts, United States of America; 6 Guttmann Brain Health Institut, Institut Guttmann, Universitat Autonoma Barcelona, Spain; 7 Department of Neurology, Harvard Medical School, Boston, Massachusetts, United States of America; Ghent University, BELGIUM

## Abstract

Several decades of research suggest that weak electric fields may influence neural processing, including those induced by neuronal activity and proposed as a substrate for a potential new cellular communication system, i.e., ephaptic transmission. Here we aim to model mesoscopic ephaptic activity in the human brain and explore its trajectory during aging by characterizing the electric field generated by cortical dipoles using realistic finite element modeling. Extrapolating from electrophysiological measurements, we first observe that modeled endogenous field magnitudes are comparable to those in measurements of weak but functionally relevant self-generated fields and to those produced by noninvasive transcranial brain stimulation, and therefore possibly able to modulate neuronal activity. Then, to evaluate the role of these fields in the human cortex in large MRI databases, we adapt an interaction approximation that considers the relative orientation of neuron and field to estimate the membrane potential perturbation in pyramidal cells. We use this approximation to define a simplified metric (EMOD1) that weights dipole coupling as a function of distance and relative orientation between emitter and receiver and evaluate it in a sample of 401 realistic human brain models from healthy subjects aged 16–83. Results reveal that ephaptic coupling, in the simplified mesoscopic modeling approach used here, significantly decreases with age, with higher involvement of sensorimotor regions and medial brain structures. This study suggests that by providing the means for fast and direct interaction between neurons, ephaptic modulation may contribute to the complexity of human function for cognition and behavior, and its modification across the lifespan and in response to pathology.

## Author summary

We study the potential role of a less understood type of communication between neurons in the brain. While the principal mechanism for neuron communication is synaptic, active neurons generate electric fields. Whether this is physiologically relevant at the systems level or merely an epiphenomenon is uncertain because these fields are rather weak at the mesoscopic scale (i.e., between single neuron and entire brain scales). We first review converging evidence from in-vitro and in-vivo studies that suggest the former, and then, using realistic finite element modeling, we show that the electric fields generated by transcranial electrical current stimulation are of the same magnitude as endogenous ones in this scale. We then develop a method to estimate the amount of potential ephaptic or electric-field interaction in an individual brain using finite element modeling and show its decrease with age in a large cohort of 401 subjects. Ephaptic interaction may be important for complex processing in biological neural networks, because it travels at very fast speeds and provides a potential communication link across distant neurons in the cortex. Assessing the physiological relevance of this mechanism may be key in understanding some brain disorders and to design improved tES protocols.

## Introduction

Jefferys [1] defined population electric field effects as those “in which the synchronous activity of populations of neurons causes large electric fields that can affect the excitability of suitably oriented, but not closely neighboring, neurons”. The literature refers to these, loosely, as “ephaptic interactions”. Traveling at the speed of electromagnetic radiation, self-generated or endogenous (ephaptic coupling) fields provide the means for fast and direct interaction between neurons, enabling new mechanisms for communication and computation that remain incompletely understood. Although much faster than chemical synaptic transmission and with a longer range than electrical synaptic communication in gap junctions (a few nm [2]), electromagnetic waves travel slower in biological media than in vacuum. Table A in S1 Text summarizes the relevant electromagnetic properties of tissues in the brain, including propagation velocity.

Work in the last decades has shown that neuronal circuits are surprisingly sensitive to weak endogenous or exogenous low frequency (0–100 Hz) electric fields (> 0.1 V/m). For example, Frohlich et al [3] showed that exogenous direct current (DC) and low frequency alternating current (AC) electric fields modulate neocortical network activity in slices with a threshold of 0.5 V/m. They also found effects from the application of exogenous fields mimicking endogenous fields recorded from the slices. More recent research has further established the role of ephaptic interactions and the sensitivity of neuronal populations to weak fields both in-vitro and in-silico. In particular, it demonstrates that endogenous fields are capable of mediating the propagation of self-regenerating slow (∼0.1 m/s) neural waves [4,5] and that externally applied extracellular electric fields with amplitudes in the range of endogenous fields are sufficient to modulate or block the propagation of this activity both in vitro and in silico models [6]. Field amplitudes in the range of 0.1–5 V/m have also been shown to produce physiological effects in primates using transcranial electrical current stimulation (see, e.g., [7] for recent results in nonhuman primates). Table B in S1 Text provides an overview spanning six decades of in-vivo and in-vitro research on the physiological impact of weak, low frequency (< 100 Hz) electric fields—both exogenous and endogenous.

Here we focus on endogenous fields that may contribute to short-range communication at or above millimeter scales, that is, not ultra-local ephaptic effects coupling adjacent neurons (see, e.g., references in [8] or those in [9]), i.e., mesoscopic scales at intermediate scales of the nervous system, between single neurons and the entire brain [10]. That is, inspired our work in transcranial current stimulation (tES) modeling, we are interested in the characterization of electric fields in the brain at mesoscopic scales similar to the ones of relevance in tES. This in contrast with other relevant ephaptic field research, which concentrates on the microscopic (single neuron) scale. Our approach, which complements it, stems from the intuition that if tES macroscopic fields have physiological effects, it makes sense to ask the question in the context of ephaptic interaction at the same spatiotemporal scales, which can be guided by EEG phenomenology.

The generation of fields capable of effectively bridging such distances requires the synchronized activity of neuronal populations [11,12] radiating from cortical patches, which occurs at frequencies below about 100 Hz (the “EEG regime”) and with spatial correlation scales in the order of a centimeter. We will call these slow, mesoscopic endogenous electric (ephaptic) fields SMEFs for short. As SMEFs appear to be of physiological relevance (v. Table B) and not simply an epiphenomenon, understanding how and where they play a functional role may be necessary for the development of realistic models of neural dynamics and function. Additional motivation for this study derives from seeking a theory for the effects of the weak exogenous electric fields—such as the ones generated by transcranial electrical current stimulation (tES or tES, as it is sometimes known). At the frequencies of interest here (*<*100 Hz), both endogenous electric fields and exogenous tES fields are characterized by relatively large spatial correlation scales (of the order of centimeter or more) and low magnitudes (*>* 0.1 V/m). Gaining a better understanding of ephaptic effects may shed some light on how tES modulates neural dynamics and, eventually, how to optimize it.

First, we use modern biophysical modeling tools to characterize macroscopic endogenous fields (i.e., spatially averaged at linear scales *>*0.1 mm, *v*. [13], section 4.3) using realistic finite element method (FEM) head modeling. In the Methods section, we describe how we model the electric fields from EEG generating cortical populations at experimentally observed densities and patch sizes and compare them with those described in available experimental work. We analyze this in an idealized analytical model (S1 Text), in a simple 3D “toy” model, and, finally, in a realistic brain model derived from an individual MRI.

Based on this, we propose an **ephaptic modulation index** that can be computed on individual from realistic brain models (EMOD) to characterize ephaptic coupling in an individual’s brain and a derive a first simplified version for computational convenience (EMOD1). Although existing metrics such as gyrification, cortical thickness or surface area capture some geometric aspects relevant to ephaptic coupling, we take a more physics-grounded approach. We build on existing models for the interaction of mesoscopic weak electric fields and neurons as used in the field of transcranial current electrical stimulation (the “lambda-E” model [14]). Considering the cytoarchitecture of the cortex placing pyramidal cells oriented perpendicular to the cortical surface, the lambda-E model indicates that the quantity of relevance to study electric field effects is the normal or orthogonal component of the field to the cortex (*E*_*n*_).

Finally, we analyze how EMOD1 changes across the lifespan by characterizing it from individual structural MRIs of a large sample of 401 heathy individuals aged 16–83. EMOD1 and structural morphologies such as cortical thickness, surface area and gyrification, were correlated with age, providing a map of brain regions whose potential for ephaptic transmission, as described by our model, is significantly affected by aging. Such findings suggest further research, and in particular in computational model studies (Sanchez-Todo et. al., 2018, Ruffini et al. 2018), to understand whether ephaptic modulation might have relevance for cognitive processing and for the manifestation of pathological conditions involving brain morphometric changes as well alterations of oscillatory patterns (e.g., schizophrenia [15], depression [16], Alzheimer’s Disease [17] or Parkinson’s [18]).

## Materials and methods

### Mechanisms

Given their anatomical characteristics (elongated form factor, which enhances the effects of electric field on membrane polarization), organization (horizontal connectivity, homogeneous orientation in cortical patches and temporal synchrony [12]), cortical pyramidal cells are well suited as electric field generators [12]. In analogy with reciprocity principles that apply to electromagnetic radiation antennae, for the same reason they are good field sensors of quasi-static (endogenous or exogenous) electric fields. Other cortical neuron types, however, may also play a role depending on their form factor and other characteristics. For example, perturbations of interneurons have been shown to be necessary in some modeling studies [19], and it has been argued that glial cells may undergo polarizations of c. 2 mV under tDCS which may be further amplified a columnar arrangement similar to pyramidal cells [20].

tES (also known as tCS) is a family of noninvasive techniques that include direct current (tDCS), alternating current (tACS), random noise current stimulation (tRNS) or others using specially designed waveforms. It consists in the delivery of weak current waveforms through the scalp (with electrode current intensity to electrode contact area ratios of about 0.3–5 A/m^2^) at low frequencies (0–1 kHz) resulting in weak but spatially extended electric fields in the brain (with amplitudes of about 0.1–2 V/m) [14]. tES is applied during several minutes (typically *∼*20 minutes). Such electric fields alone cannot initiate action potentials, but they can influence the likelihood of neuronal firing by the modulation of neuronal transmembrane potentials in relatively large cortical patches, resulting in changes in firing rates and spike timing [21,22]. The sustained application of such weak fields during sufficiently long periods of time (several minutes) leads to plastic changes of neuronal connectivity through Hebbian mechanisms (see, e.g., [23–25]). Thus, like SMEFs, the main characteristics of exogenous tES macroscopic fields are that they are weak, low frequency with moderate to large spatial correlation scales (*>* 1 cm), and, in practice, applied for relatively long times.

The acute or concurrent effects of tES are understood to be mediated by the coupling of electric fields to ordered populations of elongated neurons, especially pyramidal cells (see [14,26] and references therein). Neurons are influenced mostly by the component of the electric field parallel to their trajectory [3,27–30], and, therefore, knowledge about the orientation of the electric field is crucial to predict the effects of stimulation. The components of the field perpendicular and parallel to the cortical surface are of special importance since pyramidal cells near the cortical surface are mostly aligned perpendicularly to the surface, while many cortical interneurons and axonal projections of pyramidal cells tend to align parallel to the surface [31–33]. For a long, straight finite fiber with space constant λ in a homogeneous electric field ***E***, the transmembrane potential difference is largest at the fiber termination, with a value that can be approximated to first order by

**Fig 1:**
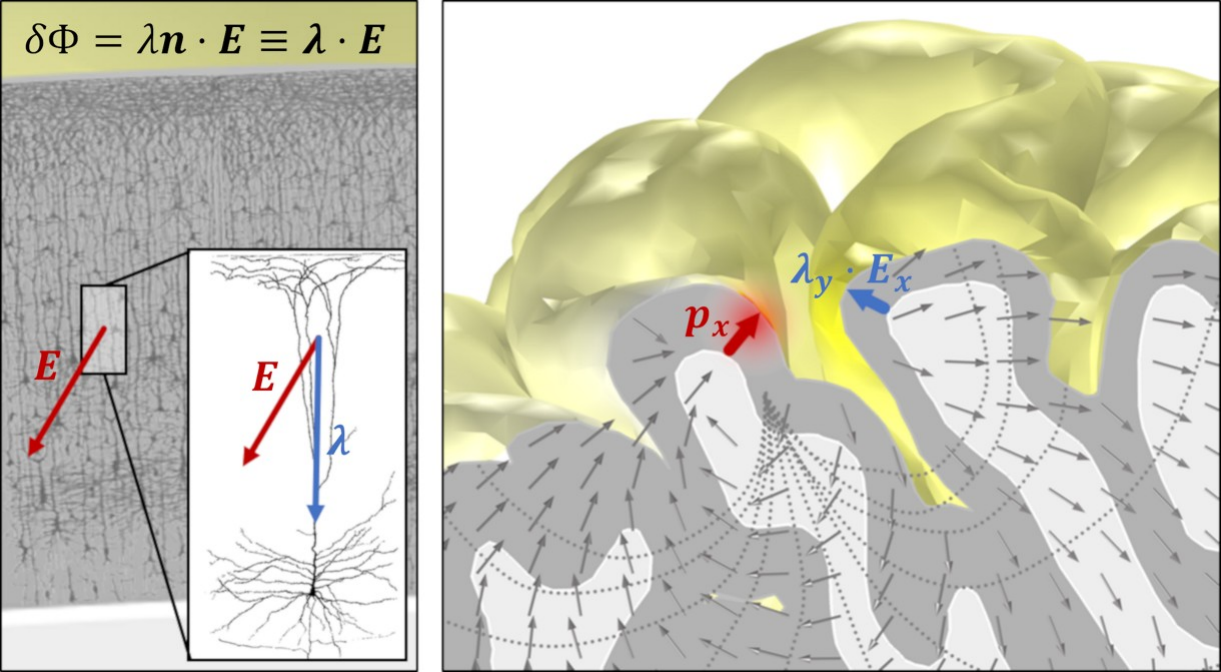
First order model for interaction of electric fields with elongated neurons. On the left, pyramidal neuron population from the human cortex (edited from “Comparative study of the sensory areas of the human cortex” by Santiago Ramon y Cajal, published in 1899, Wikipedia Public Domain). On the right, realistic model of the electric field generated by a current a dipole located at *x* in the cortex. The orientation of the generating dipole or neuron population and the sensing population (at point *y*) both play a role.

δΦ=λn∙E≡λ∙E, (1)

where *n* is the unit vector defining the fiber axis in the orthodromic direction (see Fig 1). We take this as the starting point of an approximation for the effect of a homogeneous electric field on a neural mass, which is sometimes called the “lambda-E model” [14,34] (but see also [35,36]), where the spatial scale is defined by an “effective” neuron space constant representing a neural mass average (mean field). The effect is modulated by the relative orientation of field and elongated neuronal populations. Note that an important assumption is that the electric field at the receiver site is spatially homogeneous and applies at the neuronal mass level as an approximation to more detailed models (see, e.g., [37]). The effective membrane perturbation effect is thus determined by both field magnitude and by its direction with respect to the neuronal population.

Although we do study this here, it is surprising how weak perturbations can affect neural dynamics. Membrane perturbations from weak fields are sub-threshold (about 0.1–0.2 mV per V/m applied [26]—significantly lower than the 20 mV depolarization required to bring a neuron from resting potential to spike threshold in vitro [38]). One important aspect is the large-scale nature of such perturbations, affecting large cortical patches in a uniform manner. Nonlinear effects in coupled populations are believed to underlie an amplification of these effects. For example, mathematical models have demonstrated the amplification of weak but coherent signals in networks of nonlinear oscillators (see, e.g., [39,40] and, more specifically, in computational models of neural circuits [3,4]). This effect is ultimately dependent on the coupling strength of network elements and their architecture, while noise can contribute to the enhancement of small but homogeneous perturbations in the network (array enhanced stochastic resonance [40]). Thus, cooperative effects arising from noise and coupling in coupled systems can lead to an enhancement of the network response over that of a single element. The theory of critical phenomena may also play an important role in this regard. That the brain exhibits characteristics of criticality, and therefore large sensitivity to perturbations, that may be modeled in paradigms like the Ising model is now well established, with ideas that go back to pioneers such as Turing, Bak [41] and Hopfield [42]. There is further evidence that the dynamics of the healthy brain occupy a sub-critical zone (see [43] and references therein). In fact, these two mechanisms, stochastic resonance and criticality, are probably closely related in the context of the human brain [44,45]. Similar amplification mechanisms could also play a role in other phenomena where a surprising sensitivity to weak perturbations has been found, as with the effects of Earth-strength magnetic field rotations in EEG alpha band activity [46].

In summary, assemblies of neurons, if appropriately and homogenously oriented, can function as antennae for ephaptic coupling. We adopt here the lambda-E model to estimate ephaptic effects, given the similar features of exogenous and endogenous fields of interest.

### Simplified 3D volume conductor model of ephaptic interactions

**Fig 2:**
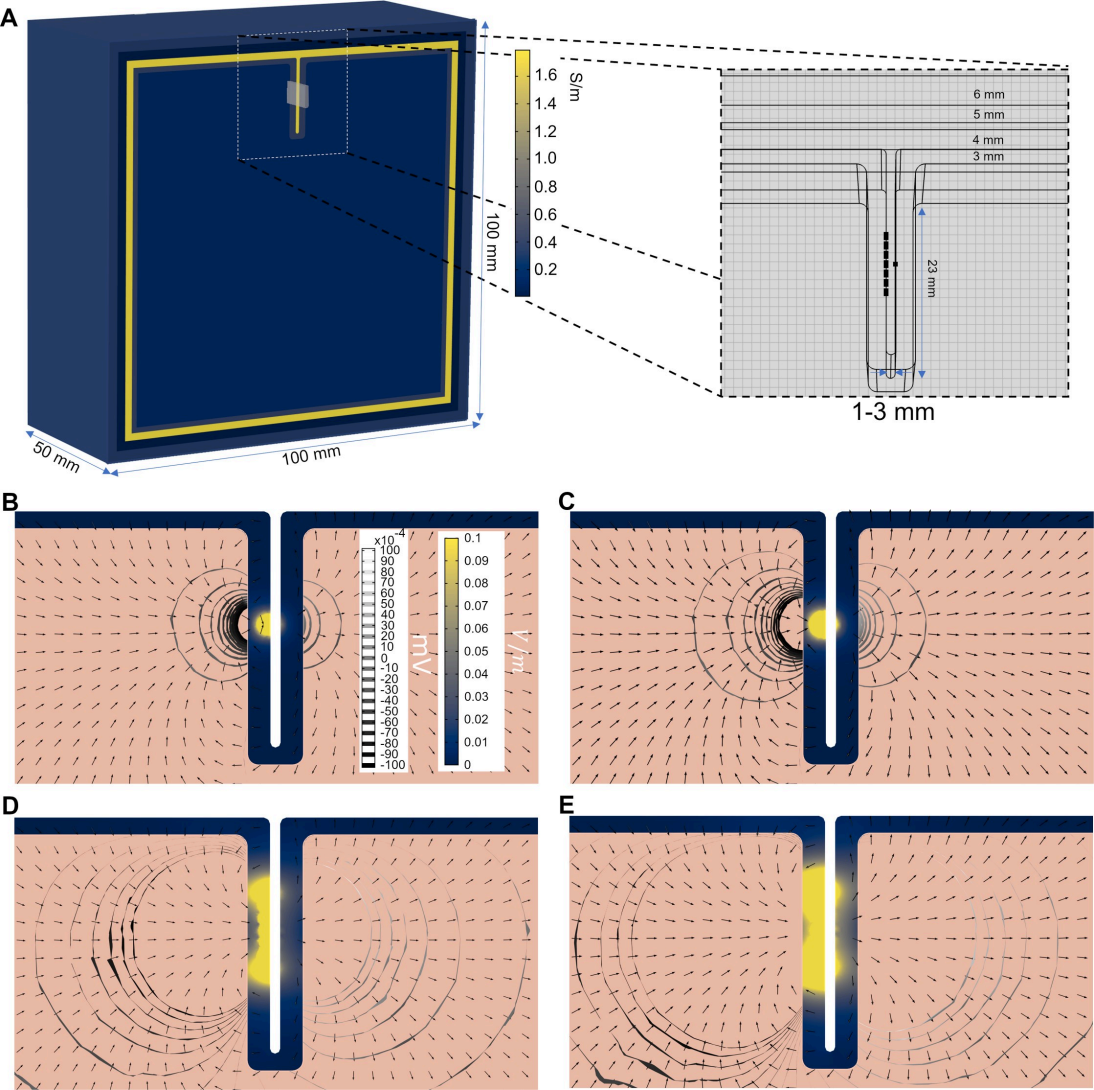
Geometry and electric field distribution in a simplified model of a sulcus. (A) 3D view of half of the simplified volume conductor (100×100×100 mm). The different tissues are colored by their respective conductivity, in S/m. The patch of single dipole sources is placed in the central region of the model (posterior wall of the sulcus), covering an area of 60 mm^2^. The figure’s inset shows a sagittal view of the model (sulcus width of 1 mm) with dipole sources in its posterior wall. (B-E) Magnitude of the electric field in the GM tissue for models with different source strength and patch distributions (common color scale between plots in V/m). Also shown are vector plots of the electric field and isosurfaces of the electrostatic potential. Left/right columns represent the models with the sources scaled to a density of 0.5 and 1.0 nAm/mm^2^ respectively. Top/bottom rows represent multiple/single dipole distributions. The colorscale is saturated to 0.1 V/m to better show the E-field in the sulcal wall opposite to the location of the sources. The E-field in the sulcus wall with the sources is much higher (1 order of magnitude higher), but its distribution is not the focus of this work.

Considerations stemming from the reciprocity theorem (see S10 Fig) indicate that potentially spatially spread dipoles of the order of 100 *nA*∙*m* are necessary to generate scalp EEG measurements of a few μV (see S1 Text). This is consistent as a result of coherent activity in cortical patches of a few square centimeters that would also generate sizeable electric fields at least up to a few mm from the source. To investigate in more detail the electric field distribution created by dipole sources on a heterogeneous volume conductor and the effects of geometric parameters such as sulcus width, we first created a 3D finite element *toy* model. The model, shown in Fig 2, includes a simplified representation of a sulcus and of the scalp, skull, cerebrospinal fluid (CSF), grey- matter (GM) and white-matter (WM) tissues. This geometry was then extruded 100 *mm* along the z-axis (out of plane direction). Sources were placed in a patch located in the posterior wall of the sulcus, in the GM-CSF interface.

The tissues were assumed to be homogeneous and isotropic, with electrical conductivity values appropriate to the low frequency range of interest [35,47]: 0.33, 0.008, 1.79, 0.40 and 0.15 *S*/*m* respectively for scalp, skull, CSF, GM and WM. Sources were modeled as point dipoles, with a direction perpendicular to the sulcus wall. Two models for the sources were built: a single dipole model and a multiple dipole model with the sources scaled to a density of either 0.5 or 1.0 nAm/mm^2^ (77 dipoles located in a 1 *mm* × 1 *mm* regular grid comprising a 60 *mm*^2^ patch—as shown in Fig 2), consistently with measurements in the human neocortex indicating that current dipole surface densities in the cortex are in the range of 0.16–0.77 *nA*∙*m*/*mm*^2^ [48,49]. The single dipole model was used to study the electric field distribution of a dipole source and its decay with distance. The multiple dipole model was used as a more realistic representation of a patch of sources. For each source model, the sulcus width was varied between 1 and 3 mm, which are median sulcus width values on the low/high-end of the reported sulci width for subjects between 20 and 80 years of age [50]. All models were solved in Comsol with the AC/DC package (v5.3a, www.comsol.com). This software solves for Laplace’s equation subject to continuity boundary conditions (continuity of the electrostatic potential and normal component of the current density) at the internal interfaces of the model. Dipole sources were modeled with Comsol’s “Electric Point Dipole” boundary condition, which allows the user to specify the direction and strength of the dipole. These sources are implemented as a contribution to the weak formulation of the problem in Comsol. The finite element mesh comprised tetrahedral second order Lagrange elements with a minimum size in the GM and CSF layers of 0.5 mm.

### Realistic brain model of endogenous fields derived from MRI

**Fig 3:**
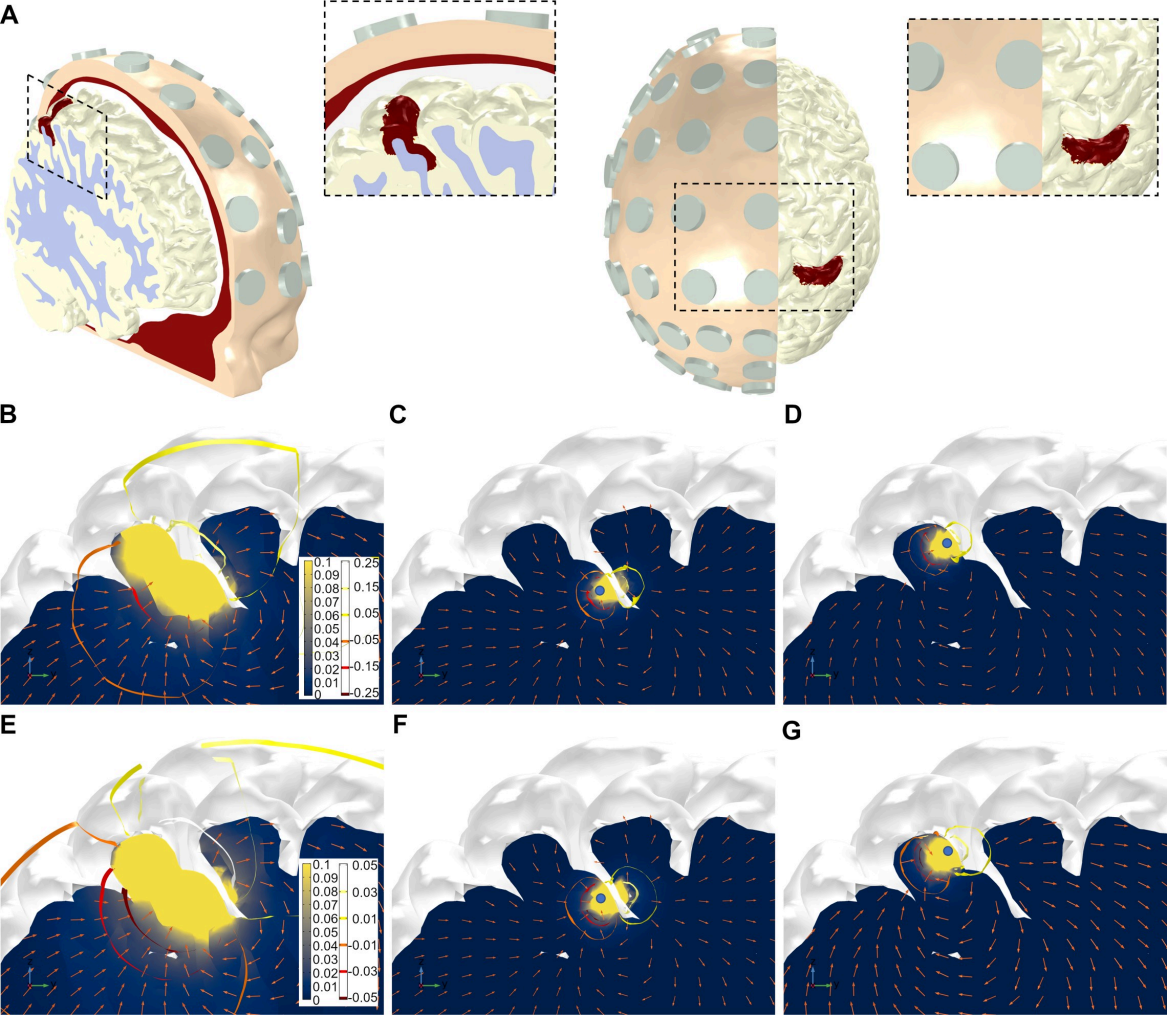
Realistic head model. (A) Two views of the 3D volume conductor geometry, including volumes representing the scalp (yellow), skull (red), CSF (white), GM (light-grey) and WM (light blue). Models of electrodes, placed in the 10–10 EEG positions, are also included in the model (grey). The patch used to place dipoles in the multiple-source model (posterior wall of the post-central sulcus, on the right hemisphere) is displayed in red in the GM volume. It comprises a cortical surface of 5.30 cm^2^. The captions provide zoomed views of the cortical patch with the dipole sources. (B-G): Electric field magnitude (color bar in V*/*m) and vector field direction, and isosurfaces of the electrostatic potential (mV) in a sagittal slice passing through the middle of the right hemisphere post-central sulcus. First (B-D) and second (E-F) rows: dipole density per unit area of 0.5*/*1.0 nAm*/*mm^2^. Columns, from left to right: model with all dipole sources, model with single dipole in narrow region of the sulcus, model with single dipole in wide region of the sulcus. The location of the individual dipoles in the middle and right-most columns are shown as blue circles in Figs C and D. The sulcus is approximately 5.5 mm wide in its wide region and 1.8 mm wide in its narrow region. For the same reasons as highlighted in Fig 2, the colorscale of the E-field's magnitude is saturated to 0.1 V/m.

The electric fields generated in the brain with tES can be readily modeled at the individual level using imaging data (MRI, see [34,51] for recent reviews). We employ here the same techniques to model endogenous fields from cortical dipoles, that is, finite element modeling derived from MRI (see Fig 3). The model, described in detail in [35], is based on the Colin27 MRI dataset (http://www.bic.mni.mcgill.ca/ServicesAtlases/Colin27). It includes realistic representations of the scalp, skull, CSF (including ventricles), GM and WM. The boundary conditions of the problem as well as the electrical properties of the tissues were similar to those described in the last section. Dipole sources were placed in the grey matter-cerebrospinal fluid (GM-CSF) surface of the model, perpendicularly to it, in similar fashion to what was done in the 3D simplified model. As before, two source distributions were calculated: a single node source mode and a multiple source model comprising a cortical surface of 5.30 cm^2^. The purpose of the single dipole models was to investigate the decay of the E-field with distance in several locations across the cortical surface. In these, the cortical surface was parcellated into 112 AAL areas and a point was chosen randomly in each area, for a total of 112 single source models. The multiple source model was built by placing 133 dipole nodes in the posterior wall of the post-central sulcus (see Fig 3B). All electric field calculations were performed in *Comsol* with the AC/DC package.

### Ephaptic modulation index (EMOD and EMOD1)

In this section we define an index to estimate, for a given individual brain model, the role of ephaptic modulation at mesoscopic scales. The index provides an average over the cortex of the impact that emitting dipoles have on receivers, with both emitter and receivers seen at the mesoscopic scale. We have considered several aspects to define it meaningfully. First, it should reflect the basic physics of dipoles (field decay with distance) and coupling of large scale fields with neurons (directional lambda-E model [14]). Second, we wish to design it to be insensitive to local effects of a dipole on its local neighbors on the cortical manifold, as this will be a strong but unspecific effect, in the sense that it will have a strong impact on the metric (from the decay with distance) while not reflecting individual anatomical variability. Rather, we would like to emphasize the effects of neighboring dipoles across-sulcus. Finally, for ephaptic effects from near dipoles to add to some relevant value, they should be *coherent* in time. This means the metric should disregard remote sources (e.g., a few cm away), which will be rapidly increase in number while becoming less coherent (the coherence length scale of cortical patches is of a few cm). The coherence space scale in the cortex depends on the frequency of the dynamics of interest. For instance, the spatial correlation length of dipole activity in the cortex is larger at lower frequencies. It is often stated that a coherent patch of 6 cm^2^ is needed to create signals that can be detected by EEG [11]. It is partly for these reasons that EEG power is weaker at high frequencies (there is no frequency dependence on conductivity at the frequencies of interest, as discussed in [52]). This also indicates that ephaptic effects are probably frequency dependent, and stronger at low frequencies.

Now, using the lambda-E tES interaction model, the ephaptic impact of a source dipole at y on a neuron or neuron population receiver at x (in μ*V*) may be approximated by *ε*_*y*_(*x*) = ***λ***_***x***_∙***E***_***y***_(*x*), where ***E***_***y***_(*x*) is the endogenous electric field vector at *x* generated by a dipole at *y* and ***λ***_***x***_ the space constant vector of the receiver neuron or neuronal population at x. The membrane perturbation may be positive (depolarizing) or negative (hyperpolarizing).

We sum ephaptic the contributions from dipole generators over the cortical mesh surface (all *y ≠ x) to* produce a total ephaptic impact factor for each cortical location *x* is (in *μ*V),

$$\varepsilon \left(x\right)=\sum_{y\neq x}W\left(x,y\right)\varepsilon_{y}\left(x\right)$$ (2)

where *W* (*x*,*y*) is a support function to be chosen to account for the requirements of non-local but coherent (not too distant) contributions. This is a local measure on the cortical surface, which we can use to produce cortical surface maps of ephaptic effects.

In the same vein, the average global index equation for a cortex is simply (μ*V*):

$$\varepsilon^{g}=\frac{1}{N}\underset{x}{\sum }\varepsilon \left(x\right)=\frac{1}{N}\underset{x}{\sum }\underset{y\neq x}{\sum }W\left(x,y\right)\varepsilon_{y}\left(x\right)$$ (3)

with N the number of nodes in the cortical mesh.

While Eq 3 provides a generic, precise expression (EMOD), it is hard to compute in practice (a realistic head model of cortical dipole electric field at each node needs to be evaluated). Given a dipole ***p*** at location *x*, what is the associated ***E*** at some nearby point *y*? As a first approximation, the electric field from a current dipole in a homogeneous conductive medium is (in polar coordinates, see [53], p. 33):

$$E=-\nabla \Phi =\frac{1}{4\pi \sigma }p\cdot \nabla \left(\frac{1}{r}\right)=\frac{1}{4\pi \sigma }\frac{p}{r^{3}}(\sin\theta \;\hat{\theta }+2\;\cos\theta \hat{r})$$ (4)

where *r* is the distance between *x* and *y*, and *σ* the conductivity of the medium. With receiver neuron mass on “top” of the dipole (at *θ* = 0), *ε*_*y*_(*x*) becomes

$$\varepsilon_{y}(x)=\lambda_{x}\cdot E_{y}(x)\approx \frac{2}{4\pi \sigma }\frac{\lambda_{x}\cdot p_{y}}{r^{3}}$$ (5)

We will set ***p***_*y*_ = *p*_0_*δA****n***_*y*_ with *p*_0_ = 0.5*nA*∙*m*/*mm*^2^ and ***λ***_*x*_ = *λ*_0_***n***_*x*_ with *λ*_0_ = 1*mm*. We denote the local unit normal vector at the source at *y* by ***n***_*y*_. We collect some of these factors into a constant for use below, *κ* = *λ*_0_*p*_0_/(2*πσ*) (with conductivity evaluated at GM). Based on this, we provide a simplified approximation which uses the fact that dipole strength falls, approximately, as the cube of the distance, with ***n***_*x*_ and ***n***_*y*_ denoting local unit cortical surface normal vectors at source and receiver locations,

$$\varepsilon \left(x\right)\approx -\kappa \underset{y\neq x}{\sum }W\left(x,y\right)\frac{n_{x}\cdot n_{y}}{r^{3}}\delta A$$ (6)

(the minus sign accounts for the opposing direction of surface normal and space normal vector conventions used here, with ***λ***_*x*_ pointing in the orthodromic direction). This index takes into account orientation of dipole and affected populations, and in particular, if the effect of the dipole on other regions is excitatory or inhibitory. Finally, to select contributions from near dipoles in Euclidean space but geodesically distant on the surface (e.g., across sulci with opposed orientation), we write

$$\varepsilon_{1}\left(x\right)\approx -\kappa \underset{y\neq x}{\sum }\Theta \left[-n_{x}\cdot n_{y}\right]\Theta \left[l_{0}-r\right]\frac{n_{x}\cdot n_{y}}{r^{3}}\delta A$$ (7)

and

$$\varepsilon_{1}^{g}\approx -\frac{\kappa }{N}\underset{x}{\sum }\underset{y\neq x}{\sum }\Theta \left[-n_{x}\cdot n_{y}\right]\Theta \left[l_{0}-r\right]\frac{n_{x}\cdot n_{y}}{r^{3}}\delta A$$ (8)

that is, with the weighting term *W* (*x*,*y*) = Θ[−***n***_*x*_∙***n***_*y*_]Θ[*l*_0_−*r*], with Θ[*x*] the Heaviside step function (defined as Θ[*x*] for *x*≤0 and 1 otherwise) and *l*_0_ a scale relevant for interaction (maximal distance to consider coherent contributions). We set *l*_0_ = 5 *mm*.

We call this simplified index EMOD1 (see S1 Text for a discussion on variants of EMOD1). It can be computed vertex-wise to produce cortical maps or averaged over the surface. Its calculation requires only the segmentation of the cortical surface and calculation of surface normal vectors from MRI images. In this formulation we implicitly assume a uniform distribution of the area of the triangles in the cortical surface mesh. This is in practice not the case, as regions with higher surface curvature tend to have a higher density of elements (therefore smaller areas). By not explicitly including the areas in EMOD1’s expression, we increase the weights that those regions have on the calculation. In addition to reinforcing the role of sulcal regions (which benefit the most from an increase in mesh density), this may, however, also be plausible from a physical point of view, as regions of high curvature will result in higher E-field values (Bhattacharya, 2016), a detail that our model does not account for explicitly.

### Imaging data and analysis

To test the variation of the ephaptic modulation index with age, we calculated it (using the simplified expression in Eqs 7 and 8) for 401 subjects with ages between 16–83 years using a publicly available database. High-quality structural T1-weighted MRIs (3T) were acquired for 401 subjects from the NKI-Rockland database [54]. MRI images were acquired using a 3-T Siemens MAGNETOM TrioTim with the following parameters: MPRAGE sequence, TR = 1900ms, TE = 2.52ms, and TI = 900ms, Flip Angle = 9 degrees, FOV = 250x250mm, voxel size = 1 mm isotropic.

Structural T1-weighted MRIs were processed using the Freesurfer v6.0 software package to create three-dimensional representations of cortical surface [55]. The Freesurfer pipeline includes automated Talairach transformation, segmentation of subcortical white matter and deep grey matter structures based on intensity and neighbor constraints, intensity normalization, tessellation of grey matter-white matter boundary and grey matter-CSF boundary, automated topology correction and reconstruction of cortical surface meshes [56]. Next, reconstructed white surfaces were registered to Freesurfer template (*fsaverage*) based on cortical folding patterns using spherical registration implemented in Freesurfer (*mri surf2surf*).

For each subject, we also have computed cortical morphometrics including cortical thickness, surface area, and gyrification. Gyrification quantifies the cortical surface hidden in the sulci as compared to the visible cortical surface. The vertex-wise cortical gyrification was measured by calculating the gyrification index in circular three- dimensional regions of interest [57]. This method uses an outer smooth surface tightly wrapping the pial surface and computes the ratio between areas of circular regions on the outer surface and their corresponding circular patches on the pial surface (see https://surfer.nmr.mgh.harvard.edu/fswiki/LGI for a description of how to calculate it with Freesurfer). At each vertex, cortical thickness was measured as the distance between white and pial surfaces, and cortical surface area was calculated by averaging the area of all faces that meet at a given vertex on the white matter surface.

Spherical registration implemented in Freesurfer (mri surf2surf) was used to register white matter surfaces into *Freesurfer* common template (*fsaverage*) to perform group-level analyses. We used 10 mm full-width-at-half-maximum (FWHM) Gaussian kernel to smooth cortical thickness, surface area, gyrification and EMOD1 maps.

### EMOD calculation

For EMOD1 calculation, the GM meshes obtained from Freesurfer were corrected from morphological defects using the *Mayavi* (https://docs.enthought.com/mayavi/mayavi/) and *Pymeshfix* (https://pypi.org/project/pymeshfix/) toolboxes for *Python*. Surface normal vectors were then calculated in Matlab (v2018a, www.matlab.com) using the Iso2Mesh pipeline (http://iso2mesh.sourceforge.net/cgi-bin/index.cgi). For each mesh point of the surface we also calculated the Euclidean distances to all the other points in the mesh, and used this information to compute EMOD1 locally and then globally using Eqs 7 and 8.

### Statistical analysis

Statistical analysis of correlations of metrics with age has been carried out using the Pearson correlation coefficient and its associated statistical significance using the Student’s t-distribution. All regressions were performed with the *Statsmodels* package for Python [58].

We performed vertex-*wise* Pearson’s correlation analyses between EMOD1 and cortical morphologies (cortical thickness, surface area and gyrification) as well as subjects’ age. False discovery rate (FDR) approach was used to control for multiple comparisons (Benjamini-Hochberg procedure, corrected p-value *<* 0.05) [59].

## Results

### Ephaptic map from cortical patch sources in simplified 3D model

Median sulcal width in human brains across the age span can vary between 0.5 and 5 mm [50]. Using this as a reference, we first studied the characteristics of endogenous fields in a 3D toy model of a sulcus in the cortex. The electric field distribution in the simplified 3D models for a sulcus width of 1 mm is shown in Fig 2 for the multiple dipole model (middle row, Fig 2C and 2D) and the single dipole model (bottom row, Fig 2E and 2F). Dipole strength in the multiple dipole model was set to 0.39 and 0.78 nAm, which results in a dipole strength density per unit area of 0.5 and 1.0 nAm/mm^2^ in the modeled 60 mm^2^ cortical patch. The dipole strength in the single source model was set to the same value, which results in a physiologically realistic [48,49] local density of 0.5 and 1.0 nAm/mm^2^ in the equivalent area associated to this dipole (60 and 77 mm^2^). As can be seen in the figure, in the models with the higher dipole density (1.0 nAm/mm^2^), an electric field >0.1 V/m can be observed in the wall opposite to the one where the sources are located. This is observed in both the multiple and single source models, although, as expected, the area in which the electric field is greater than 0.1 V/m is higher in the former than in the latter (the electric field from multiple-source patches decays much slower than the single dipole source case [11], p. 37). This effect was only observed in the model with sulcus width of 1 mm. Increasing the sulcus width led to lower electric field values on the opposite sulcal wall. S1 Fig in S1 Text displays the decay of the normal component of the electric field and the electrostatic potential with distance. The decays of *V* and *E*_*n*_ are well fit by a power function with exponents of −0.66, −0.88 and −2.11, −3.02, respectively, for the multiple source and single source models.

### Ephaptic map from cortical patch sources in realistic head model

Next, we analyzed the electric fields in a realistic head model. For each one of 112 single dipole models, we calculated the decay of *E*_*n*_ with Euclidean distance to the source. For all models, the decay was well fit by a power function, with an exponential of *−*3.2*±*0.8 (*R*^2^ of fit was 0.76*±*0.12). Comparing the decay of the normal component of the electric field with distance in the cortical surface, we see that it is approximately monotonic for the Euclidean distance, as expected, but not for the geodesic distance (see S1 Fig in S1 Text, bottom). This behavior is expected and a result of surface folding. For the multiple dipole source patch model, different configurations were tested using 133 dipole node sources, with individual dipole strengths adjusted so that the dipole strength area density was of 0.5 nAm*/*mm^2^ or 1.0 nAm*/*mm^2^. This resulted in individual dipole strengths at each node between 1.9 and 4.0 nAm. We also calculated single node dipole versions of these models, with strengths of 2.1 and 4.2 nAm, which correspond to the same density values in the equivalent (mesh triangle) patch size covered by that dipole. For these source strengths, it is possible to achieve an electric field magnitude of at least 0.1 V*/*m on the opposite sulcus wall (see Fig 3). This effect is local and dependent on the distance between source and sulcus wall. Using single source models positioned in the narrow part of the sulcus (Fig 3B–3E) and in the wide part of the sulcus (Fig 3C–3F) we found that only the former induced a 0.1 V/m electric field on the opposite sulcus wall. These results mimic closely those observed in the simplified volume conductor model discussed previously, since for the chosen study area sulcus separation in the realistic model was 1.4–5.5 mm in the dipole patch region (see S2 Fig in S1 Text). For reference, sulcus width in the human cortex can be less than 1 mm [50]. Table 1 summarizes the maxima of the electrostatic potential (at scalp level) and the electric field in the GM for all the realistic head the models presented here. See also S9 Fig for the scalp potential map associated to the chosen dipole patch.

**Table 1:** Summary of the maximum values of the scalp electrostatic potential (*V*) and GM electric field (magnitude, *E*, and normal component, *E*_*n*_) induced in all the source distributions used in the realistic head model. For each quantity, two dipole densities are considered: 0.5 and 1.0 nAm*/*mm^2^.

Number of dipole sources	Dipole strength area density (*nA*∙*m*/*mm*^2^)		Individual dipole strength (*nA*∙*m*)		*V*_*Scalp*_(*μV*)		Electric field in GM (V*/*m)			
							*E*		*E*_*n*_	
133	0.5	1.0	1.9	3.8	15.9	31.8	8.3	16.7	8.1	16.2
1 (narrow part of the sulcus)	0.5	1.0	2.1	4.2	0.1	0.1	1.6	3.1	1.6	3.1
1 (wide part of the sulcus)	0.5	1.0	2.1	4.2	0.3	0.5	0.3	0.7	0.3	0.7

Finally, as a check of the realistic model, we investigated the voltage distribution at the scalp induced by a single source dipole on the chosen cortical area with a strength of 100 nAm, which is what reciprocity considerations predict would be required to achieve ~10 μV at scalp level (see Methods). The dipole was aligned to the electric field induced in that node by a montage with CP2 as the anode (1 mA) and T10 as the cathode (*−*1 mA). The potential difference between electrodes CP2 and T10 was of 13 μV (within the expected bounds of the approximation).

### Ephaptic modulation in the human brain

**Fig 4:**
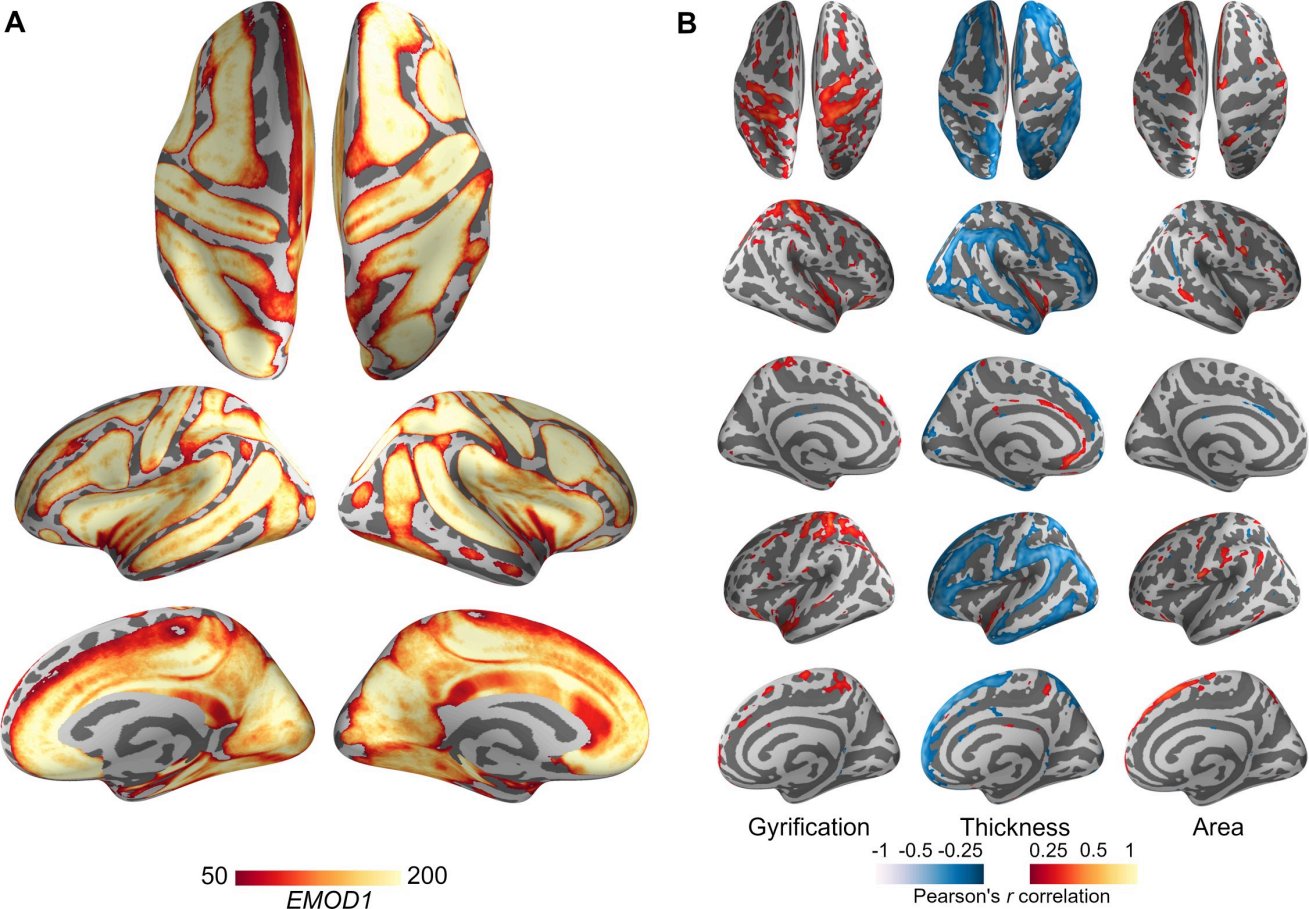
Ephaptic Modulation in the human brain. (**A) Average EMOD1.** Individual EMOD1 maps are registered to Freesurfer’s common template (*fsaverage*) and then averaged at each vertex across subjects. For the purpose of visualization, we have thresholded the average EMOD1 map at EMOD1>50. (**B) Vertex-wise correlation.** At each vertex, the Pearson’s correlation coefficient between EMOD1 and cortical surface area, thickness, gyrification and subject’s age is computed. The resulting maps are then corrected for multiple comparisons using the Benjamini- Hochberg procedure (p-value *<*0.05). Pearson’s correlation coefficient values for vertices that passed the multiple comparison correction are overlaid on Freesurfer common template (*fsaverage*).

In order to provide a template map for the distribution of ephaptic modulation in the human brain, as well as for its aging-related trajectory, 401 structural MRIs of healthy participants aged 16–83 yrs. were processed using Freesurfer software, obtaining vertex-wise cortical thickness, surface area and gyrification LGI maps for each brain. Pial surfaces obtained via Freesurfer were then used to calculate ephaptic modulation using the EMOD1 coefficient (Eq 8 with *l*_0_ = 5*mm*). A first average ephaptic map was obtained by averaging the resulting 401 EMOD1 maps (Fig 4A, and S4 Fig). As expected, following cortical gyrification patterns, the topography of EMOD1 displayed higher values along the sulci walls as well as medial regions such as the precuneus, and anterior cingulate cortex (see figures for statistical results).

In order to understand the relationship between ephaptic and other cortical morphologies (i.e., cortical thickness, surface area, gyrification), vertex-wise correlation was performed between EMOD1 and each morphological metric (Fig 4B). EMOD1 displayed significant but spatially different correlations with all the three morphologies, suggesting the magnitude of ephaptic modulation as potentially resulting from different cortical, non-exclusive structural patterns. EMOD1 also displayed a positive correlation with gyrification and surface area, and a negative correlation with cortical thickness following sulcal patterns (Fig 4B).

### Changes in Ephaptic modulation with aging

**Fig 5:**
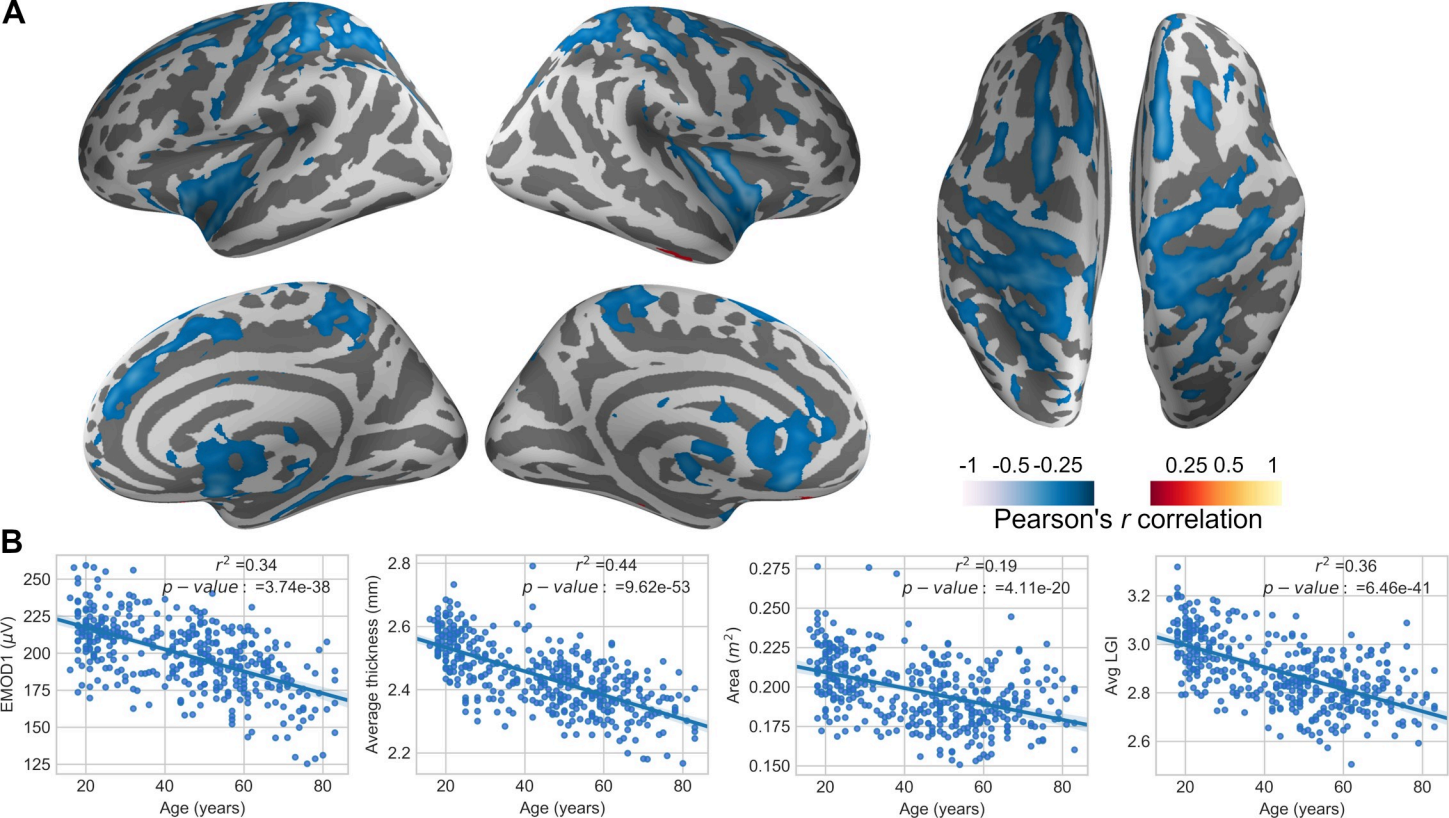
EMOD1, thickness, Area and LGI–correlation with age. (A) Vertex-wise EMOD1 values were correlated with age across the sample of 401 subjects, resulting in a weighted map displaying the cortical regions whose ephaptic modulation index is significantly affected by aging. (B) Individual data for correlation between age, EMOD1, as well as cortical morphologies are displayed. Red-yellow shows positive and blue-cyan negative correlations.

Vertex-wise correlation between EMOD1 and age produces a bilateral pattern involving primarily sensorimotor regions, insular cortex and anterior cingulate cortex (Fig 5A). The same correlation was performed for thickness, gyrification and surface area. Globally, all metrics show a tendency to decrease with age (see also S3–S6 Figs). The decrease is very well approximated by a linear function for the EMOD1, average LGI and average thickness metrics, with *R*^2^ values of the linear fits of 0.34, 0.36 and 0.44, respectively. All of these fits are statistically significant, with p-values of 3.7 *×* 10^*−*38^, 6.5 *×* 10^*−*41^ and 9.6 *×* 10^*−*53^, respectively. For the total cortical area, the fit is worse (*R*^2^ of 0.19) but still statistically significant (p- value of 4.1 *×* 10^*−*20^). Pearson-correlation coefficients between EMOD1 and average LGI/thickness are also relatively high (0.52 and 0.43, respectively, as shown in S7 and S8 Figs).

## Discussion

Understanding the functional role of ephaptic mechanisms can, among others, shed new light on the mechanisms underlying neuronal oscillations or help drive the design of better brain stimulation solutions. Research can be guided by focusing on the main features of ephaptic interactions: very fast, bidirectional, propagation of information (see Table A) between cortical sites, influencing both local and synaptically distant regions as long as they are near in (3D) space, and in a direction dictated by the state and orientation of the emitting and receiving populations (i.e., with effects that can be both excitatory and inhibitory). For example, ephaptic interaction may play an important role in cortical recurrent computation, providing the means for fast integration of information across areas with impact at both low and high frequencies. This may be especially important for gamma synchronization, where timing requirements are stringent [60]. On the other hand, ephaptic interaction has been shown to enable the generation and propagation of slow waves in brain slices–even after they have been split [6]. Similarly, SMEFs could play a role in inter-hemispheric communication, bypassing corpus callosum connections. Other recent work suggests that they could play a role in the modulation of release of extracellular vesicles [61], a newly discovered form of cellular communication.

Relying on biophysical modeling and high-resolution neuroimaging analysis, we have built a first metric of mesoscale ephaptic interaction in the human brain, characterizing its spatial distribution and its relationship with aging. Below we discuss the implications of such large scale ephaptic coupling in humans, including their potential relevance for regulating brain oscillatory patterns and cortical excitability, their evolutionary meaning as well as potential role in neurological and pathological disorders.

### Insights from models

Modeling results confirm many of the assumptions of the theoretical predictions. On the one hand, the decay of the electric field created by single dipole sources is confirmed to be well approximated by a 1*/r*^3^ power law, even in models that consider tissue heterogeneity. In the realistic model, multiple dipole sources create a field that decays slower (1*/r*^2^), as predicted by the 3D simplified sulcus model. This confirms that ephaptic interactions are limited to regions that are located close to one another. In the case of sulci, this limits interactions either to the cells close to the source(s) along the same wall, or cells on the opposite sulcus wall. We note that if the cortical region of interest is undergoing synchronous oscillations in a given band, the ephaptic effects will be in phase for dipoles along the same wall, and antiphase on the opposite wall. In our models with dipole density of 1.0 nAm*/*mm^2^, and assuming that the threshold for interaction was 0.1 V*/*m, ephaptic effects on the opposite sulcus wall could only be observed in the 3D toy model when the sulcus width was of 1 mm or less, and in the realistic 3D model in portions of the post-central sulcus where its width was the smallest (about 1.4 mm). For comparison, in Chiang et al. [6], a separation greater than 0.4 mm in a cut hippocampus slice was sufficient to impede ephaptic wave propagation (see Table B), which, together with other findings, supports our selection of an analysis threshold of 0.1 V/m.

Further evidence that the scaling of the sources in these models is realistic comes from the observation that the maximum electrostatic potential recorded at scalp level in the realistic head model varied between 16 and 32 μV, respectively for a dipole density of 0.5 and 1.0 nAm*/*mm^2^. Since these dipoles comprise a cortical area of 5.3 cm^2^, these results seem consistent with the rule of thumb that *∼*6 cm^2^ of activated cortical area are needed to produce detectable EEG at scalp level [11].

### Topography of endogenous fields in the human brain

As we have seen, EMOD1 is related to other metrics such as gyrification and cortical thickness. The latter is hardly surprising, since cross-sulcal ephaptic interaction requires the presence of cortical folding. The current study may provide further clues into the importance of gyrification as a zero-order proxy for ephaptic interaction. Studies have indicated that cortical gyrification is strongly and positively related to cortical volume but negatively related to cortical thickness in many regions of the cortex, and that frontal gyrification is positively related to performance in working memory and mental flexibility tasks [62,63]. Such results support the view that greater cortical gyrification is related to bigger brain volumes and better cognitive function. One advantage of gyrification is thought to be increased speed of brain cell communication, since cortical folds allow for cells to be closer to one other, requiring less time and energy to transmit neuronal electrical impulses [49]. Ephaptic interactions and EMOD1 reflect similar advantages. As can be observed in Fig 4A, the ephaptic hotspots as determined by EMOD are quite pronounced. This suggests the study of neural dynamics in such locations as a function of the synchronization level of activity in surrounding areas, under the hypothesis that hotspots should be especially sensitive to changes in cross-sulcal synchronization.

From an evolutionary point of view, we may hypothesize that natural selection forces that promoted folding the cortex to fit a larger cortical surface in a more static cranium (i.e., cortical gyrification), as a byproduct made available ephaptic interaction as a form of information transfer, which then also underwent natural selection. Across species, the degree of cortical folding correlates with brain weight and, more specifically, with cortical surface area. In all major mammalian lineages, the species with large brains tend to have more highly folded cortices than species with smaller brains (v. [64] and references therein). The pilot whale and bottlenose dolphin display the highest gyrification index values. The human brain, while larger than that of a horse, shows a similar gyrification index. Rodents generally display the lowest gyrification. Nonetheless, some rodents show gyrencephaly and a few primate species are quite lissencephalic. Research on the evolutionary biology studying ephaptic transmission is deeply needed.

### Ephaptic coupling and age

Analysis of the metrics computed on the MRI dataset indicate a robust correlation of EMOD1, cortical thickness, LGI, and surface area with age, as displayed in Fig 5. Not surprisingly, these metrics display moderate inter-correlations stemming from the covariation of cortical folding and sulcal separation. The index proposed here, which stems from physiological considerations related to ephaptic coupling, relies strongly on the notion of sulcal width and dipole strength (cubic) decay with distance. Studies of sulcal widening have shown it is associated to aging, decreased cognitive ability, dementia and schizophrenia [50]. The negative association observed between EMOD1 and age suggest a highly speculative yet interesting scenario, where the decrease of ephaptic coupling with age may contribute to loss of control over oscillatory patterns and cortical excitability, potentially contributing to age-related cognitive changes. Furthermore, pathologies associated with cortical atrophy, e.g., dementia or traumatic brain injury, would alter ephaptic transmission as well, contributing to the pathophysiology as well as cognitive and behavioral symptoms.

Related to age-related changes in brain structures, the concept of “brain age” has been recently explored by multiple groups, looking at how structural MRI data can be used to estimate the “actual” biological age of a given brain as compared to his chronological age [65–67]. Such analysis is carried out by fitting a model estimating chronological age by means of structural MRI data in a sample of age matched participants, to then compare residual values for each participant and label each brain as respectively “older” or “younger” than its reference cohort. Interestingly, estimated brain age has been shown to correlate with mortality, making a very interesting novel health biomarker [66]. The structural properties such as LGI, thickness and grey matter density are considered, but no studies have investigated the potential role of ephaptic coupling distribution in determining brain age. Together with other potential mechanisms, such as functional reallocation of fMRI connectivity patterns, ephaptic coupling might constitute another key element to determine and maintain brain age.

### Ephaptic role in neurological disorders

Hypersynchronized activity in seizure can generate large rhythmic fields of 20–70 V/m in the hippocampus and 3–9 V/m in the neocortex (v. [68]). Interictal discharges generate strong ephaptic perturbations that might very rapidly alter brain dynamics and cause, or at least contribute to, their deleterious effects on brain function and cognition, as also discussed in [4]. Interestingly, cortical malformations of various types, including shallow sulci and defects of cellular migration, have been described in epilepsy as well [69], possibly linking cortical morphology and aberrant epileptic activity through alterations of ephaptic transmission.

More specifically, ephaptic interaction might play a role in the pathogenesis of seizure via its potential contribution to self-regulation of cortical excitability. As the cortical walls come in close proximity due to cortical folding, by projecting activity with the opposite phase on neighboring areas, ephaptic interaction might protect the brain from hypersynchronization. By the same token, the increasing amplitude and spatial extent of electrical activity generated during the last stage of a seizure (see, e.g. [70]) may act, through ephaptic interaction, as a homeostatic mechanism to end the seizure. Interestingly, focal cortical dysplasia lesions associated with epileptiform activity are preferentially located at the bottom of abnormally deep sulci [71], where such ephaptic homeostatic control would be weakest for geometric reasons.

Alteration of ephaptic interaction can also shed new light on other human brain disorders that are accompanied by change in cortical gyrification. For instance, Lissencephaly is a rare, genetically related brain malformation characterized by the absence of normal convolutions in the cerebral cortex and an abnormally small head. Symptoms may include unusual facial appearance, difficulty swallowing, failure to thrive, muscle spasms, seizures, and severe psychomotor retardation. Laminar heterotopia is a rare condition consisting in an extra layer of gray matter underlying properly migrated cortex, usually associated with epileptiform activity, cognitive deficits and alterations of functional connectivity patterns [72,73]. Polymicrogyria is a condition in which the brain has an overly convoluted cortex. Symptoms can include seizures, delayed development or weakened muscles. Higher levels of gyrification are also found to relate to greater local connectivity in the brains of individuals with autism spectrum disorders, suggesting ephaptically mediated hyperconnectivity [74]. The same could be predicted of healthy populations: increased ephaptic coupling (LGI and EMOD) would be associated to increased functionally connectivity, especially at high frequencies. Similarly, the brains of patients with schizophrenia also show reduced cortical thickness and increased gyrification when compared to healthy brains [75]. Further studies on ephaptic transmission in various pathologies may offer novel insights to account for the identified alterations in brain oscillations and explain cognitive and behavioral symptomatology.

### Relationship between tES and ephaptic coupling

Together with in-vitro and animal work demonstrating the physiological effects of weak electrical perturbations, abundant work in recent years indicates that weak electric fields applied over relatively large areas and over a duration of minutes can have significant physiological after-effects in humans [76]. Interestingly, as highlighted above endogenous fields are of the same order of magnitude as those generated by tES, and both display large correlation scales (of the order of centimeters). In addition, in both types of electric fields are present in the cortex for relatively long times (minutes in tES and indefinitely with endogenous fields), and, at the scales of interest, at relatively low frequencies (<< 1 kHz). These similarities suggest that the neuromodulatory effects of tES may rely on a natural brain interaction mechanism.

For example, it is likely that the effects of tES, which generates electric fields of the order of 0.1–2 V/m (as predicted by models and verified experimentally [77,78]) may ultimately be explained by “spatiotemporal coherence” mechanisms, that is, to the augmented impact of weak but spatially extended, temporally coherent (DC or AC) and persistent (minutes) electric fields [14,79] on neuronal networks in the presence of background noise. Such “array enhanced” emission and reception features would apply to both exogenous and endogenous fields.

A consequent question is how we can use these insights for better design of tES protocols. If tES leverages a natural and physiologically relevant ephaptic mechanism, understanding it in detail should provide valuable inputs for the design of optimized tES in disorders such as epilepsy, depression or neuropathic pain, where questions remain on where to apply electric fields, for how long and with what temporal waveforms (DC, AC or endogenous, e.g., as derived from EEG), or, perhaps, to help understand what distinguishes treatment responders from non-responders. In particular, the design of tES protocols should be conceived from the point of view of generating a summation of endogenous and exogeneous fields which the cortex will interact with as an endogenous one. For example, if age or atrophy (e.g., in dementia) predict a reduced impact of ephaptic interactions, would this also suggest a decrease of response to tES? The hypothesis here would be that a brain that has lost the ability to engage in ephaptic communication will similarly be less sensitive to the effects of exogenous fields.

### Limitations of the study and future directions

The conclusions drawn from our electric field models are subject to uncertainties in some parameters that may affect the volume conduction effects of the currents induced by the dipole sources. Some of these parameters are the conductivity properties of the tissues in the head in the low-frequency range of EEG. These conductivity values are known to considerably influence the electric field distribution in the brain, but the reported range of values in the literature is still somewhat inconsistent [80]. They are also known to vary with individual anatomy, age and disease [81–84]. Other important parameters in the model are dipole density and patch size. These are of critical importance, since they influence the location and size of the areas which are influenced by source activity.

An important limitation in this study is the use of a simplified metric (EMOD1) as opposed to a full calculation of the ephaptic field generated by cortical dipoles (EMOD proper, Eq 4) and the sensitivity of this metric to a particular weighting scheme. Our physics approach represents a convenient trade-off to be able to evaluate this metric on a large dataset, and it should certainly be improved in the future.

Still related to the calculation of EMOD1, in this formulation we implicitly assume a uniform distribution of the area of the triangles in the cortical surface mesh. As discussed in the Methods section, this is in practice not the case, as regions with higher surface curvature tend to have a higher density of elements (therefore smaller areas). By not explicitly including the areas in EMOD1’s expression, we increase the weights that those regions have on the calculation. This may, however, be plausible from a physical point of view, as regions of high curvature will also result in higher E-field values [85], a detail that our model does not account for explicitly. In fact, we have observed that by not including such an area correction the correlation of EMOD1 with age increases significantly (from 0.07 to 0.34). Future work should disentangle the role of curvature and pure mesh density from a biophysical point of view to better justify this choice.

In addition, and equally importantly, we used here an interaction model that does not consider the complexity or spatial distribution of pyramidal neurons, or the effects on other types of neurons, or the complexities associated to the dynamical nature of neural physiology– much as it is done in brain stimulation research, with some justification [14,19,34] for the analysis of tES effects. Only recently the effects of tES have been studied in computational models of the brain [19,86,87] using the lambda-E model discussed above, but yet ignoring the intricacies of micro-cortical network circuitry. Our modeling work and EMOD inherits all these limitations: this is a first approach that will be improved in the future.

Further work remains to be carried out to disentangle the differential contributions of EMOD1, cortical thickness and other cortical morphologies to explaining measures of brain function and cognition. An interesting line of research will be to determine computationally the impact of ephaptic interaction on neuronal dynamics in both the healthy and pathological cortex, along the lines proposed in [34].

## Conclusions

Our findings, in line with earlier experimental work, provide additional information to assess the relevance of ephaptic transmission for an improved understanding of brain function and human cognition, as well as neurological and psychiatric pathology where brain structural alterations are present.

## Supporting information

S1 Text Additional information about the assumptions underlying the derivation of EMOD1 and its correlations with other metrics. (DOCX)

S1 Fig Decay of *V* and *E*_*n*_ in the 2D and 3D models of the sulcus. Top: field decay in 2D model. (A) Decay of *V* with sulcus width in the single source model (blue dots) and multiple sources model (orange dots). The fit to a power function is also shown for each model. (B) Same as (A), but now for ***E***_***n***_, the component of the electric field normal to the sulcus wall. (C) Field decay in 3D model: l*oglog* plot of |***E***_***n***_| (in V*/*m) in the GM-CSF surface as a function of the logarithm of the geodesic (blue dots) or Euclidean (red dots) distance (in mm) to the dipole. The inset shows ***E***_***n***_ (in V*/*m) in a 3D rendering of the cortical surface. The location of the source is indicated by the red arrow. Only points where the absolute value of ***E***_***n***_ is between 0.001 V*/*m and 1.0 V*/*m are shown. Linear fits to these plots are also shown, together with the slope and *R*^2^ values.
(TIF)

S2 Fig Sulcus geometry. Measurements of width (mm) in the sulcus used for realistic modeling in Fig 3 in the main text. Note that this is an easy to compute approximation (bounded from above) to the minimal distance between sulcal wall points.
(TIF)

S3 Fig Surface distribution of the EMOD1 coefficient (*l*_0_ of 5 mm) for subjects with different ages. Subjects are presented from highest (top) to lowest EMOD1 (bottom) values. The color scale is common across all the plots. From left-right: top/bottom view, left/right-hemisphere view, front/back view, mid sagittal place left/right hemisphere view.
(TIF)

S4 FigLinear fits of EMOD variants to age.Different rows correspond to different EMOD1 variants: EMOD0 ($\varepsilon_{0}^{g}$), EMOD1a ($\varepsilon_{1a}^{g}$) and EMOD1 ($\varepsilon_{1}^{g}$). Different columns correspond to different l0 parameters: 1, 5, 10 and 200 mm, respectively from left to right.(TIF)

S5 FigSecond order fits of EMOD variants to age.Different rows correspond to different EMOD1 variants: EMOD0 ($\varepsilon_{0}^{g}$), EMOD1a ($\varepsilon_{1a}^{g}$) and EMOD1 ($\varepsilon_{1}^{g}$). Different columns correspond to different *l*_0_ parameters: 1, 5, 10 and 200 mm, respectively from left to right.(TIF)

S6 Fig Second order fits of EMOD1, average LGI, average cortical thickness and cortical area to age. For each plot, r-squared and p-values for the fit are shown as well.
(TIF)

S7 Fig Pearson correlation coefficients between different EMOD variants, average LGI, average cortical thickness and total surface area. (TIF)

S8 Fig Correlation between average LGI, EMOD1 (*l*_0_ set to 5 mm), average cortical thickness and total cortical area for different age range groups. The plots along the main diagonal show histograms of these quantities grouped by age range. The offline range elements show each variable plotted against all others. Pearson correlation coefficients for each pairing, divided by age group, are also presented.
(TIF)

S9 Fig EEG (referenced to T8, in μV) as generated by cortical patch in Fig 3 (see also Table 1). The dipole patch consists of 133 dipole sources (patch area of 5.3 cm^2^), with a dipole density of 0.5 nAm/mm^2^.
(TIF)

S10 Fig Illustration of reciprocity theorem for a bipolar montage. Consider a hypothetical reciprocal EEG measurement where we observe a potential difference *V*_*ab*_ between the same points *a* and *b* produced by a dipole *p* located at *x* and normal to the cortical surface. The reciprocity theorem implies that we can replace the pair (*E*_*n*_,*I*_*ab*_) with (*V*_*ab*_,*p*) with the ratio of the first pair the same as the ratio of the second. Hence, from the current-electric field data pair we can deduce, given *V*_*ab*_, a value for a reciprocal dipole *p*: *V*_*ab*_/*p* = −*E*_*n*_/*I*_*ab*_.
(TIF)
