# Supplementary material for: Realistic modeling of mesoscopic ephaptic coupling in the human brain
Source: PLoS Comput Biol. 2020 Jun 1;16(6):e1007923. doi: 10.1371/journal.pcbi.1007923 (PMC7289436; doi:10.1371/journal.pcbi.1007923)
Supplement: S1 Text — (DOCX) [file pcbi.1007923.s001.docx]

**SUPPLEMENTARY INFORMATION**

**Realistic modeling of mesoscopic ephaptic coupling in the human brain**

Giulio Ruffini1*,*2,3*∗*, Ricardo Salvador2, Ehsan Tadayon4, Roser Sanchez-Todo2, Alvaro Pascual-Leone5,6,7, Emiliano Santarnecchi4

1 Neuroelectrics Corporation, 210 Broadway, 02139 Cambridge, MA, USA

2 Neuroelectrics Barcelona, Avda. Tibidabo, 47 bis, 08035 Barcelona, Spain

3 Starlab Barcelona, Avda. Tibidabo, 47 bis, 08035 Barcelona, Spain

4 Berenson-Allen Center for Noninvasive Brain Stimulation, Beth Israel Deaconess Medical Center and Harvard Medical School, Boston, MA, USA

5 Hinda and Arthur Marcus Institute for Aging Research and Center for Memory Health, Hebrew SeniorLife, Boston, MA, USA

6 Guttmann Brain Health Institut, Institut Guttmann, Universitat Autonoma Barcelona, Spain

7 Department of Neurology, Harvard Medical School, Boston, MA, USA

# SPEED OF ELECTROMAGNETIC WAVES IN THE BRAIN

Table A provides a summary of the speed of electromagnetic waves in brain media.

# Review of literature on the effects of slow, weak electric fields (SMEFs)

See Table B for an overview of relevant papers involving weak fields.

# Estimates of endogenous field strength from reciprocity arguments and EEG

While in the next sections we model SMEFs in the cortex using finite element models, here we provide some estimates from reciprocity considerations [1–4] by leveraging earlier work modeling the electric fields generated by tES. Realistic head modeling shows that the cortical fields associated to typical 1 mA bipolar tES are of the order of 0-0.5 V/m (electric field normal to the cortex, *E_n_*) [5], and about 5–10 times smaller when averaged over cortical patches at tES resolution scales (several cm^2^). These models have been validated by invasive measurements where a bipolar current of about 1 mA leads to median electric field magnitudes of the order of 0.1 V/m [6–8].

Reciprocity in physics, and in particular in electromagnetism, refers to a set of symmetries or invariances afforded by the formalism in which the identities of an emitter (source) and a detector (or field) can be interchanged (see., e.g., [9] ). According to the reciprocity theorem, the magnitude of the E- field normal to the cortical surface induced by a given tES bipolar montage (electrode positions and current) is proportional to the sensitivity of the same montage when used for EEG to monitor the electrical signals generated by a dipole source at the same point in the cortical surface and oriented perpendicularly to it. Let us denote by $I_{ab}=1 mA$ the current applied from point $a$ to point $b$ in the scalp that induces the cortically normal electric field *E_n_* somewhere at a point *x* in the cortex. Consider a hypothetical reciprocal EEG measurement where we observe a potential difference $V_{ab}=10 \mu V$ between the same points $a$ and $b$ produced by a dipole *p* located at *x* and normal to the cortical surface—such as the one in Fig [1.](#_bookmark28) The reciprocity theorem implies that we can replace the pair ($E_{n},I_{ab}$) with ($V_{ab},p$) with the ratio of the first pair the same as the ratio of the second. Hence, from the current-electric field data pair we can deduce, given $V_{ab}$, a value for a reciprocal dipole $p$: $V_{ab}/p = -E_{n}/I_{ab}$, which implies $\left| p \right|=\left| {I_{ab}V_{ab}}/{E_{n}} \right|={10\times{10}^{-6} V\times{10}^{-3} A}/{(0.1 V/m)}=100 nA\cdot m$ using a value from measurements of $E_{n}\approx0.1 V/m$. So, if a lone dipole located at $x$ were responsible for the observed $V_{ab}$, it would have this strength.

As an example, using the chosen realistic head model sulcus model described below (Section 5.4), we calculated the voltage distribution at the scalp induced by such a single 100 $nA\cdot m$ source dipole. The dipole was oriented normal to the cortex. At that location, the normal component of the electric field generated by a montage with CP2 as the anode (1 mA) and T10 as the cathode (*−*1 mA) was of 0.13 V/m. The potential difference between electrodes CP2 and T10 was of 13*.*1 µV, in agreement with the reciprocity calculation.

Given such a dipole $\boldsymbol{p}$ at location $x$, what is the associated $\boldsymbol{E}$ at some nearby point $y$? As a first approximation, the electric field from a current dipole in a homogeneous conductive medium is (in polar coordinates, see [3], p. 33):

|  | $\boldsymbol{E}=-\boldsymbol{\nabla}\Phi=\frac{1}{4\pi\sigma}\boldsymbol{p}\cdot\boldsymbol{\nabla}\left( \frac{1}{r} \right)$=$\frac{1}{4\pi\sigma}\frac{p}{r^{3}}\left( \sin\theta\hat{\boldsymbol{\theta}}+2\cos\theta\hat{\boldsymbol{r}} \right)$, | (s1) |
| --- | --- | --- |

where *r* is the distance between $x$ and $y$, and $\sigma$ the conductivity of the medium. For example, the field magnitude at 1 mm of distance from the idealized dipole on the contiguous cortical surface is $E \approx40 V/m$ ($\theta=0, \sigma=0.40 S/m$ in grey matter tissue, see for instance [10]). This is at the high end of DC stimulation regime experiments (in-vitro, see Table B). At 1 cm distance from the dipole, $E=0.05 V/m$. Out in the CSF, where ($\theta=90, \sigma=1.79 S/m$), the magnitudes are $E\approx4$ and $0.004 V/m$, respectively. The dipole approximation is applicable for distances significantly larger than the dipole size (the space constant of pyramidal neurons is typically much less than 1 mm, see e.g. [11]).

Of course, EEG signals are not generated by single point dipoles but by the summation of fields from extended sources (coherent patches) and collections of them. Despite of this, to the extent that these sources are small compared to scales we are interested in, these estimates give an order of magnitude of what we may expect to observe. Measurements in the human neocortex indicate that current dipole surface densities in the cortex are in the range of $0.16-0.77 nA\cdot m/{mm}^{2}$ [12,13]. There appears to be a maximum value across brain structures and species ($1-2 nA\cdot m/{mm}^{2}$). At the same time, studies using combined electrocorticography and MEG show that coherent area sizes of the order of $1$ to $20 {cm}^{2}$ are needed for MEG detection, with the larger ones observed in epileptic discharges [14]. At a density of $0.25 nA\cdot m/{mm}^{2}$, our hypothetical dipole of $100 nA\cdot m$ above would be realized over a patch of about $4 {cm}^{2}$.

In summary, considerations stemming from the reciprocity theorem indicate that dipoles of the order of $100 nA\cdot m$ are necessary to generate scalp EEG measurements of a few µV. This is consistent as a result of coherent activity in cortical patches of a few square centimeters that would also generate sizeable electric fields at least up to a few mm from the source.

Finally, we note that cortical folds bring together pyramidal populations of opposite orientation to distances of much less than $1 cm$ (even submillimeter) which should play an important role in extending the effects of dipole fields beyond their immediate neighborhoods.

1. **Decay of dipole fields**

S[1 Fig](#_bookmark28) displays plots with the decay of electric field and potential as a function of Euclidean distance for different models.

1. **3d SULCUS GEOMETRY**

S[2 Fig](#_bookmark29) displays distance measurements of the sulcus gap.

1. **EMOD1 MAPS FOR SELECTED SUBJECTS**

S3 Fig displays the surface distribution of the EMOD1 coefficient (l0 of 5 mm) for subjects with different ages.

1. **VARIANTS OF EMOD1**

We provide here some variants of EMOD1. We recall the definition of EMOD1 (with *l*0 = 5 mm):

|  | $\varepsilon_{1}^{g}\approx-\frac{\kappa}{N}\sum_{x} \sum_{y\neq x} \Theta[-\boldsymbol{n}_{x}\cdot\boldsymbol{n}_{y}]\Theta[l_{0}-r]\frac{\boldsymbol{n}_{x}\cdot\boldsymbol{n}_{y}}{r^{3}}\delta A$ | (s2) |
| --- | --- | --- |

The spatial scale *l*_0_ can be varied, but it does not have a big impact on the results.

The first main EMOD1 variant just considers the effect of distance between emitter and receiver, ignoring relative orientation:

|  | $\varepsilon_{0}^{g}\approx\frac{\kappa}{N}\sum_{x} \sum_{y\neq x} \Theta[l_{0}-r]\frac{1}{r^{3}}\delta A$ | (s3) |
| --- | --- | --- |

The second one takes into account relative orientation, but does not enforce the requirement in EMOD1 for opposite orientation of emitter and receiver (which forces cross-sulcal contributions in EMOD1):

|  | $\varepsilon_{1a}^{g}\approx\frac{\kappa}{N}\sum_{x} \sum_{y\neq x} \Theta[l_{0}-r]\frac{\left\vert\boldsymbol{n}_{x}\cdot\boldsymbol{n}_{y} \right\vert}{r^{3}}\delta A$ | (s4) |
| --- | --- | --- |

S4 Fig provides linear fits of EMOD variants with age. S5 Fig provides second order fits.

1. **SECOND ORDER CORRELATIONS OF METRICS**

S6 Fig provides second order fits of EMOD1, LGI, cortical thickness and area with to age, while S7-S8 Figs provide Pearson cross-correlation between the different metrics.

1. **SCALP MAP/EEG GENERATED BY DIPOLE PATCH MODEL**

S9 Fig displays the scalp map potential for one of the chosen dipole cortical patches (Fig 3, 0.5 nAm/mm^2^ density).

| Tissue | $\varepsilon_{r}$ | $c/v$ | $v (km/s)$ | $\tau_{20 cm} (ns)$ |
| --- | --- | --- | --- | --- |
| Vacuum | 1 | 1 | 299,792 | 0.0 |
| CSF | 109 | 10 | 28,715 | 0.0 |
| GM | 40,699,000 | 6,380 | 47 | 4.3 |
| WM | 27,627,000 | 5,256 | 57 | 3.5 |

**Table A:** Relative permittivity, speed of light reduction factor with respect to vacuum (*c/v* ), speed of light in tissue (v) in the low frequency range (around 10 Hz, where the quasi-static limit applies [15]) for various tissues, with data from [16] provided online at [http://niremf.ifac.cnr.it/tissprop/.](http://niremf.ifac.cnr.it/tissprop/) Here we use $\nu=c/\sqrt{\varepsilon_{r}\mu_{r}}\approx c/\sqrt{\varepsilon_{r}}$ (the relative magnetic permittivity in body tissues is close to unity [17]). The last column is the time in nanoseconds required by ephaptic signals to traverse a sphere of 20 cm. Speed increases 3–4 times at 100 Hz for grey matter (GM) and white matter (WM), and stays constant for cerebrospinal fluid (CSF).

**Table B:** **Overview of relevant work highlighting the physiological impact of weak electric fields in-vitro or in-vivo and providing quantitative measurements of electric field.** The range of electric field magnitude ($E=||\boldsymbol{E}||$) or of the normal component of the electric field to cell layers ($E_{n}$), as available (in V/m or, equivalently, mV/mm), that have been shown to influence function are listed. Only references where at least the magnitude of the extracellular electric field is specified are used (the voltage gradient). EPs: evoked potentials. AC: alternating current. DC: direct current. FR: firing rates. LFP: local field potential. SUA/MUA: single/multiple unit activity.

| **Reference** | **Preparation** | $\boldsymbol{\vert\vert E}$**\|\|** | $\left\vert\boldsymbol{E}_{\boldsymbol{n}} \right\vert$ | **Type** | **Effects** | **Comments** |
| --- | --- | --- | --- | --- | --- | --- |
| [18] | Abdominal receptors in the crayfish and cardiac ganglion of the lobster | 1–4 | 1 | DC | FR | Fields required were for FR changes were 20 times below threshold. Orientation dependence demonstrated. |
| [19] | Rat cortex in-vivo | 2.5 | 2.5 | DC | FR and EPs | After effects after 5–10 min stimulation were described. |
| [20] | Guinea-pig hippocampus slices | 5–70 | 5–70 | DC | EPs | Extracellular currents perpendicular to granule cell layer in hippocampal slices altered their excitability. Effects seen with fields $>$5V/m. |
| [21] | Rat hippocampus slices | 2–7 | 2–7 | AC | EPs | Brief stimulation of 5–30 s induced long term changes (more than 10 minutes) of population spike. Exogenous extracellular fields in the tissue were of the order of EEG gradients, suggesting a functional role of EEG-like fields in hippocampus. |
| [22] | Rat hippocampus slices | 0–8 | 0–8 | DC | Epileptiform activity/LFP | Modulation and full suppression of epileptiform activity was observed at field strengths between 1 and 5 V/m in a direction dependent manner. Results indicate that DC fields modulate and suppress low-calcium activity by directly polarizing CA1 pyramidal cells. |
| [23] | Rat hippocampus slices | 0.14–3.9 rms value (0.3–6.8 p-p) | same as $E$ | Simulated burst stimulus waveforms with gaussian profile | Entrainment | Neuronal networks respond to fields with more sensitivity than single neurons. Estimated theoretical lower limit for meaningful interaction between electric field and neuron is 0.1 V/m. |
| [24] | Rat hippocampal slices | 0–200 | 0–200 | DC | Membrane potential, evoked action potentials | The induced polarization was linear (0.12 $\pm$0.05 mV per V/m applied average sensitivity at the soma). DC fields altered the thresholds of action potentials evoked by orthodromic stimulation and shifted their initiation site along the apical dendrites. |
| [25] | Rat hippocampus slices | 0.5–16 | 0.5–16 | DC, AC | FR, Entrainment, Timing, Membrane potential alteration | Decreasing impact w.r.t. DC with increasing frequency. Gamma rhythms modulated by 50 Hz AC with (normal) fields $>0.5$V/m (p-p). Effects on both the power spectrum and spike timing depend on AC frequency, with slower frequencies being more effective. |
| [26] | Rat hippocampal slices | 0.5–1.0 | 0.5-1.0 | DC, AC | Timing, entrainment | Spike timing effects are a potential mechanism for the network effects of weak fields. |
| [27] | Coronal slices of ferret brain | 0–4 | 0–4 | DC, AC, in vivo-like fields, activity-dependent “feedback" fields | FR, entrainment | Enhancement of slow oscillation at its intrinsic frequency with 2 V/m, entrainment at 0.5 V/m. Significant effect at 0.5 V/m. The E field lines were approximately orthogonal to the cortical surface. |
| [28] | Rat neocortex slices (layer V pyramidal neurons) | 0.7–5.6 | N/A | AC  (1–9 Hz) | Timing | Ephaptically induced phase locking of spiking is thus more effective, and occurs at lower field strengths, for slow rather than fast modulations of $E$. $E$field as small as 0.74 V/m led to entrainment at 1 Hz. |
| [29] | In vivo, rat neocortex and hippocampus. Brain slices also analyzed. | 1 | N/A | AC  (0.8–1.7 Hz) | Entrainment | In the intact brain, neurons distant from the stimulation sites can be entrained directly through ephaptic coupling or indirectly, through multisynaptic projections of the directly entrained neurons proximal to the stimulation sites. |
| [30] | Rat hippocampus slices | 0–15 | 0–15 | DC/AC  (0–40 Hz) | Intracellular Spikes, FRs, spike timing and phase-entrainment resonance | Negative fields decreased the steady-state power of gamma oscillations measured during stimulation, positive fields increased steady-state gamma power. With fields as low as 0.2 V/m phase entrainment can occur with stimulation frequency matched to the endogenous rhythm. |
| [31] | Rat cortical pyramidal neuron slices | 0.7–4.2 | N/A | AC (1-9 Hz) | Entrainment of spikes, Timing (no FR changes) | Despite small size, fields could entrain action potentials, especially for slow ($<8$ Hz) oscillations. LFP like fluctuations readily entrain membrane potential and spiking. |
| [32] | Rat hippocampal slices | 0–60 | 0–60 | DC | Membrane polarization, spike latency and synaptic response | Significant effects on spike latency evoked by somatic current injection. The relative position and spatial orientation of dendritic trees affect both synaptic circuitry and the interaction with electric fields; subthreshold electric fields should robustly alter the balance between different rhythms, and in particular theta-gamma ratio. |
| [33] | Rat cortical brain slices | 0–8 | 0–8 | DC | field EPSPs | Polarization of both axon terminal and soma are important for effects. |
| [34] | Unfolded hippocampus preparation from mice | 3–6 | N/A | Endogenous fields | Timing | Experiments indicated that longitudinal propagation is independent of chemical or electrical synaptic transmission. Spontaneous epileptiform activity can propagate in both the transverse and longitudinal directions with a speed of 0.1 m/s independently of connectivity. |
| [35] | Mouse neocortical slices | 1–2 | 1–2 | AC | FR, Activity spectrum | Weak AC fields enhanced ongoing oscillations only if matched in frequency when strong endogenous activity was present. Enhanced activity occurred at frequency of application when no strong endogenous activity was present. Results point to the importance of frequency matching when strong endogenous oscillations are present. |
| [36] | Rat unfolded hippocampus + compartment model | 2–5 | 2–5 | DC | Reduction of propagation speed with blocking field (firing rate changes) | Results show that weak electric fields can be solely responsible for spike propagation at $\sim$0.1 m/s. This phenomenon could be important to explain the slow propagation of epileptic activity or normal propagation at similar speeds. |
| [37] | Alert nonhuman primates | 0.4–0.7 | N/A | DC | LFP, SUA/MUA in neocortex | FRs did not change but tDCS induced large low-frequency oscillations in the underlying tissue. Local increase in LFP power near the site of anodal stimulation. More wide- spread effects included a decrease in low-frequency LFP coherence between distant cortical sites along with an increase in high-frequency (gamma-band) coherence. |
| [38] | Intracellular and extracellular recordings in rats | 1–2 | N/A | AC | Membrane potential alteration. Firing rate changes. Power in delta band. | Membrane became depolarized or hyperpolarized in a relatively linear manner. Electric fields applied either subcutaneously or transcutaneously which induce at least 1 V/m voltage gradient can affect spiking activity, but stronger fields are needed to affect network oscillations. NB: Voltage gradients measured parallel to cortex, normal component probably much lower. |
| [39] | Rat motor cortex in-vivo | N/A | 1 | AC  (1–2.5 Hz) | Single neuron recording entrainment (PLV) | Weak field stimulation ($\sim$1 V/m) can entrain neural oscillations ($\sim$1 Hz) in the rat motor cortex. |
| [40] | Triple-transgenic mice used for longitudinal hippocampal slice studies | 5 | 5 | Endogenous fields and anti-fields | Propagating waves | Endogenous electric fields play a significant role in the self-propagation of slow waves ($<1$ Hz) in the hippocampus. External anti-fields can block them. Slow activity stopped propagating when cut gap was $>400\mu$m. |
| [6] | Alert nonhuman primates | 0.2–0.3 | N/A | AC  (1–100 Hz) | Single Neurons in basal ganglia & hippocampus. Spike Timing | tES consistently influences the timing, but not the rate, of spiking activity. Effects are frequency- and location-specific and can reach deep brain structures; control experiments show that results cannot be explained by sensory stimulation or other indirect influences. |
| [41] | Alert ferrets | $<$0.5 | 0.22–0.3 | AC  (6–14 Hz) | Spike-field synchrony | Weak electric fields ($<$ 0.5 mV/mm) comparable to tACS field strength in humans and nonhuman primates can entrain neural spiking in the source of target oscillations. |

REFERENCES

1. Carson JR. A Generalization of the Reciprocal Theorem. Bell System Technical Journal. 1924;3: 393–399. doi:10.1002/j.1538-7305.1924.tb00009.x

2. Helmholtz H. Uber einige Gesetz der Vertheilung elektrischer Strdme in korperlichen Leitern, mit Anwendung auf die thierisch- elektrischen Versuche. Ann Phys Chem. 1853;ser. 3, 29: 211–233 and 353–377.

3. Plonsey R. Bioelectric phenomena. McGraw-Hill; 1969.

4. Rush S, Driscoll DA. Current distribution in the brain from surface electrodes. Anesth Analg. 1968;47: 717–23.

5. Miranda PC, Mekonnen A, Salvador R, Ruffini G. The electric field in the cortex during transcranial current stimulation. NeuroImage. 2013;70: 48–58. doi:DOI 10.1016/j.neuroimage.2012.12.034

6. Krause MR, Vieira PG, Csorba BA, Pilly PK, Pack CC. Transcranial alternating current stimulation entrains single-neuron activity in the primate brain. PNAS. 2019.

7. Opitz A, Falchier A, Yan C-G, Yeagle EM, Linn GS, Megevand P, et al. Spatiotemporal structure of intracranial electric fields induced by transcranial electric stimulation in humans and nonhuman primates. Scientific Reports. 2016;6: 31236. doi:10.1038/srep31236

8. Huang Y, Liu AA, Lafon B, Friedman D, Dayan M, Wang X, et al. Measurements and models of electric fields in the in vivo human brain during transcranial electric stimulation. eLife. 2017;6.

9. Deák L, Fülöp T. Reciprocity in quantum, electromagnetic and other wave scattering. Annals of Physics. 2012;327: 1050–1077. doi:10.1016/j.aop.2011.10.013

10. Logothetis NK, al et. In vivo measurement of cortical impedance spectrum in monkeys: implications for signal propagation. Neuron. 2007;55: 809–823.

11. Aberra AS, Peterchev AV, Grill WM. Biophysically realistic neuron models for simulation of cortical stimulation. J Neural Eng. 2018;15: 066023. doi:10.1088/1741-2552/aadbb1

12. Murakami S, Okada Y. Invariance in current dipole moment density across brain structures and species: Physiological constraint for neuroimaging. Neuroimage. 2015;111.

13. Hari R, Puce A. MEG–EEG primer. Oxford U. Press; 2017.

14. Oishi M, Otsubo H, Kameyama S, Morota N, Masuda H, Kitayama M, et al. Epileptic Spikes: Magnetoencephalography versus Simultaneous Electrocorticography. Epilepsia. 2002;43: 1390–1395.

15. Plonsey R, Heppner DB. Considerations of Quasi-Stationarity in Electrophysiological Systems. B Math Biophys. 1967;29: 657–.

16. Gabriel C, Gabriel S, Corthout E. The dielectric properties of biological tissues: I. Literature survey. Phys Med Biol. 1996;41: 2231–49.

17. Schenck JF. The role of magnetic susceptibility in magnetic resonance imaging: MRI magnetic compatibility of the first and second kinds. Medical Physics. 1996;815.

18. Terzuolo CA, Bullock TH. Measurement of Imposed Voltage Gradient Adequate to Modulate Neuronal Firing. Proc Natl Acad Sci U S A. 1956;42: 687–94.

19. Bindman LJ, Lippold CJ, Redfearn JWT. The action of brief polarizing currents on the cerebral cortex of the rat (I) during current flow and (II) in the production of long-lasting effects. J Physiol. 1964;172: 369–382.

20. Jefferys JG. Influence of electric fields on the excitability of granule cells in guinea-pig hippocampal slices. J Physiol. 1981;319: 143–52.

21. Bawin SM, Sheppard AR, Mahoney MD, Adey WR. Influences of sinusoidal electric fields on excitability in the rat hippocampal slice. Brain Res. 1984;323: 227–37.

22. Ghai RS, Bikson M, Durand DM. Effects of applied electric fields on low-calcium epileptiform activity in the CA1 region of rat hippocampal slices. J Neurophysiol. 2000;84: 274–80.

23. Francis JT, Gluckman BJ, Schiff SJ. Sensitivity of neurons to weak electric fields. J Neurosci. 2003;23: 7255–61.

24. Bikson M, Inoue M, Akiyama H, Deans JK, Fox JE, Miyakawa H, et al. Effects of uniform extracellular DC electric fields on excitability in rat hippocampal slices in vitro. J Physiol. 2004;557: 175–90.

25. Deans JK, Powell AD, Jefferys JG. Sensitivity of coherent oscillations in rat hippocampus to AC electric fields. J Physiol. 2007;583: 555–65.

26. Radman T, Su Y, An JH, Parra LC, Bikson M. Spike timing amplifies the effect of electric fields on neurons: implications for endogenous field effects. J Neurosci. 2007;27: 3030–6.

27. Fröhlich F, McCormick DA. Endogenous Electric Fields May Guide Neocortical Network Activity. Neuron. 2010;67: 129–143.

28. Anastassiou CA, Montgomery SM, Barahona M, Buzsáki G, Koch C. The Effect of Spatially Inhomogeneous Extracellular Electric Fields on Neurons. J Neurosci. 2010;30: 1925–1936.

29. Ozen S, Sirota A, Belluscio MA, Anastassiou CA, Stark E, Koch C, et al. Transcranial Electric Stimulation Entrains Cortical Neuronal Populations in Rats. The Journal of Neuroscience. 2010;30: 11476–11485.

30. Reato D, Rahman A, Bikson M, Parra LC. Low-Intensity Electrical Stimulation Affects Network Dynamics by Modulating Population Rate and Spike Timing. The Journal of Neuroscience. 2010;30: 15067–15079.

31. Anastassiou CA, Perin R, Markram H, Koch C. Ephaptic coupling of cortical neurons. Nature Neuroscience. 2011;14: 17–223.

32. Berzhanskaya J, Chernyy N, Gluckman BJ, Schiff SJ, Ascoli GA. Modulation of hippocampal rhythms by subthreshold electric fields and network topology Modulation of hippocampal rhythms by subthreshold electric fields and network topology. J Comput Neurosci. 2013;34: 369–389.

33. Rahman A, Reato D, Arlotti M, Gasca F, Datta A, Parra LC, et al. Cellular effects of acute direct current stimulation: somatic and synaptic terminal effects. J Physiol. 2013;591: 2563–2578.

34. Zhang M, Ladas TP, Qiu C, Shivacharan RS, Gonzalez‐Reyes LE, Durand DM. Propagation of epileptiform activity can be independent of synaptic transmission, gap junctions, or diffusion and is consistent with electrical field transmission. J Neurosci. 2014;34: 1409–1419.

35. Schmidt SL, Iyengar AK, Foulser AA, Boyle MR, Fröhlich F. Endogenous Cortical Oscillations Constrain Neuromodulation by Weak Electric Fields. Brain Stimulation. 2015;7: 878–89.

36. Qiu C, Shivacharan RS, M MZ, Durand DM. Can neural activity propagate by endogenous electrical field? J Neurosci. 2015;35: 15800–15811.

37. Krause MR, Zanos TP, Csorba BA, Pilly PK, Choe J, Phillips ME, et al. Transcranial Direct Current Stimulation Facilitates Associative Learning and Alters Functional Connectivity in the Primate Brain. Current Biology. 2017;27: 1–11.

38. Vöröslakos M, Takeuchi Y, Fernández-Ruiz A, Buzsáki GKG, Berényi A, Brinyiczki K, et al. Direct effects of transcranial electric stimulation on brain circuits in rats and humans. Nature Communications. 2018;9.

39. Asamoah B, Khatoun A, Laughlin MM. tACS motor system effects can be caused by transcutaneous stimulation of peripheral nerves. Nature Communications. 2019;10.

40. Chiang C-C, Shivacharan RS, Wei X, Gonzalez‐Reyes LE, Durand DM. Slow periodic activity in the longitudinal hippocampal slice can self‐propagate non‐synaptically by a mechanism consistent with ephaptic coupling. The Journal of Physiology. 2019;597.

41. Negahbani E, Stitt IM, Davey M, Doan TT, Dannhauer M, Hoover AC, et al. Transcranial Alternating Current Stimulation (tACS) Entrains Alpha Oscillations by Preferential Phase Synchronization of Fast-Spiking Cortical Neurons to Stimulation Waveform. BiorRxiv. 2019.


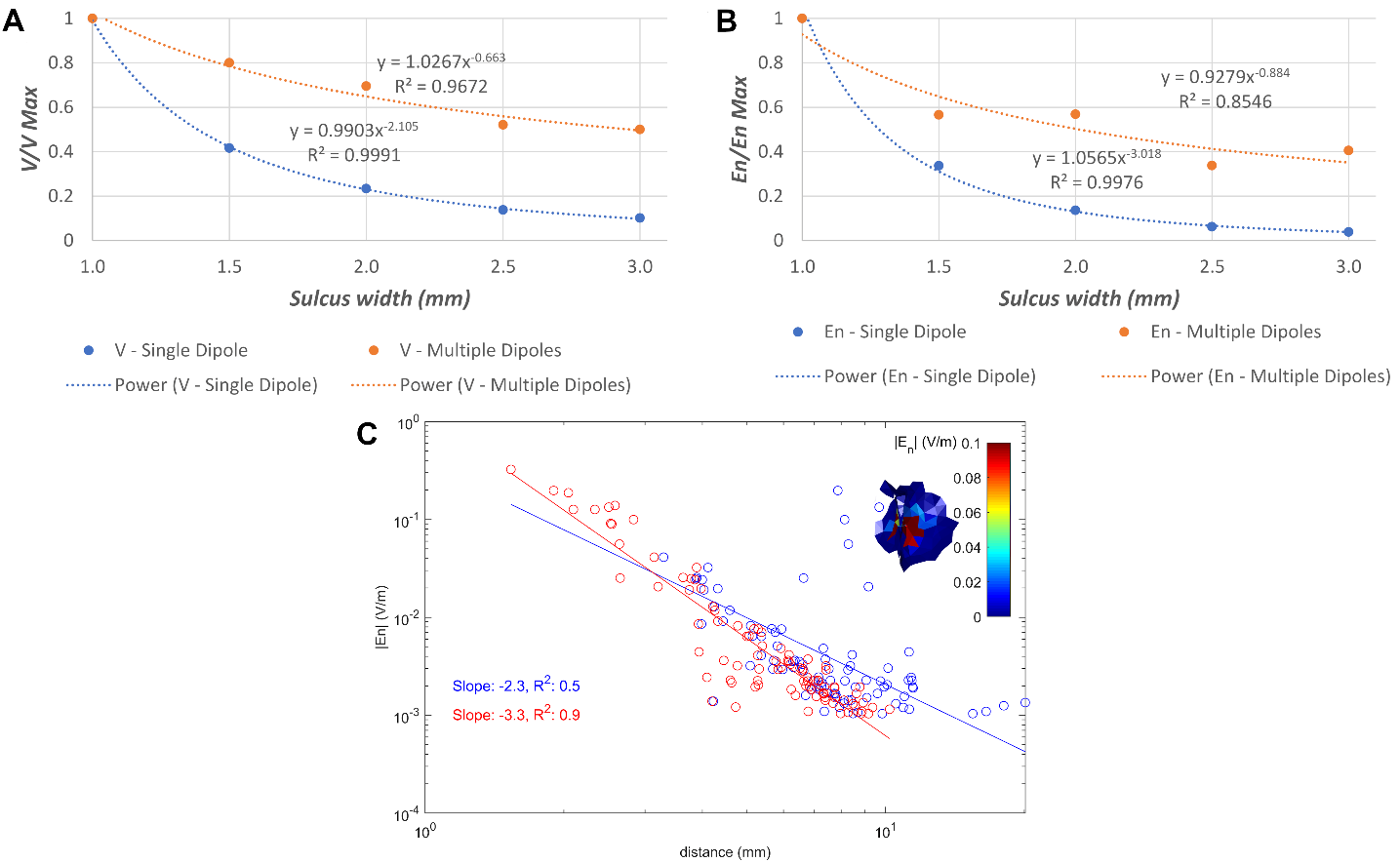


**S1 Figure:** **Decay of** *V* **and** *E_n_* **in the 2D and 3D models of the sulcus. Top: field decay in 2D model:** (A) Decay of *V* with sulcus width in the single source model (blue dots) and multiple sources model (orange dots). The fit to a power function is also shown for each model. (B) Same as (A), but now for $\boldsymbol{E}_{\boldsymbol{n}}$, the component of the electric field normal to the sulcus wall. (C) Field decay in 3D model: l*oglog* plot of $\boldsymbol{|}\boldsymbol{E}_{\boldsymbol{n}}\boldsymbol{|}$ (in V*/*m) in the GM-CSF surface as a function of the logarithm of the geodesic (blue dots) or Euclidean (red dots) distance (in mm) to the dipole. The inset shows $\boldsymbol{E}_{\boldsymbol{n}}$ (in V*/*m) in a 3D rendering of the cortical surface. The location of the source is indicated by the red arrow. Only points where the absolute value of $\boldsymbol{E}_{\boldsymbol{n}}$ is between 0*.*001 V*/*m and 1*.*0 V*/*m are shown. Linear fits to these plots are also shown, together with the slope and *R*^2^ values.





**S2 Figure:** **Sulcus geometry** Measurements of width (mm) in the sulcus used for realistic modeling in Fig 3 in the main text. Note that this is an easy to compute approximation (bounded from above) to the minimal distance between sulcal wall points.


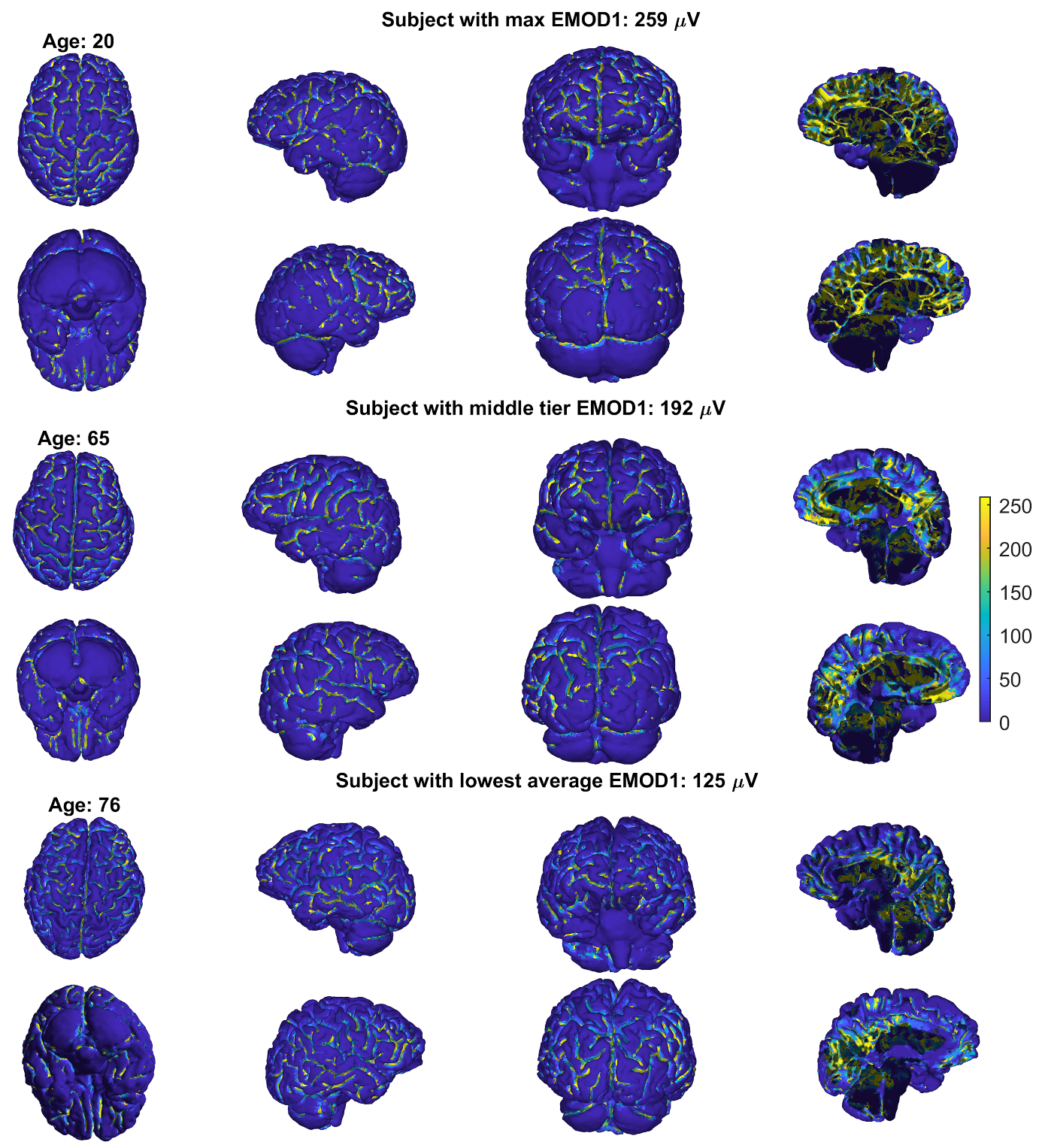


**S3 Figure:** **Surface distribution of the EMOD1 coefficient (***l*_0_ **of** 5 mm**) for subjects with different ages.** Subjects are presented from highest (top) to lowest EMOD1 (bottom) values. The color scale is common across all the plots. From left-right: top/bottom view, left/right-hemisphere view, front/back view, mid sagittal place left/right hemisphere view.


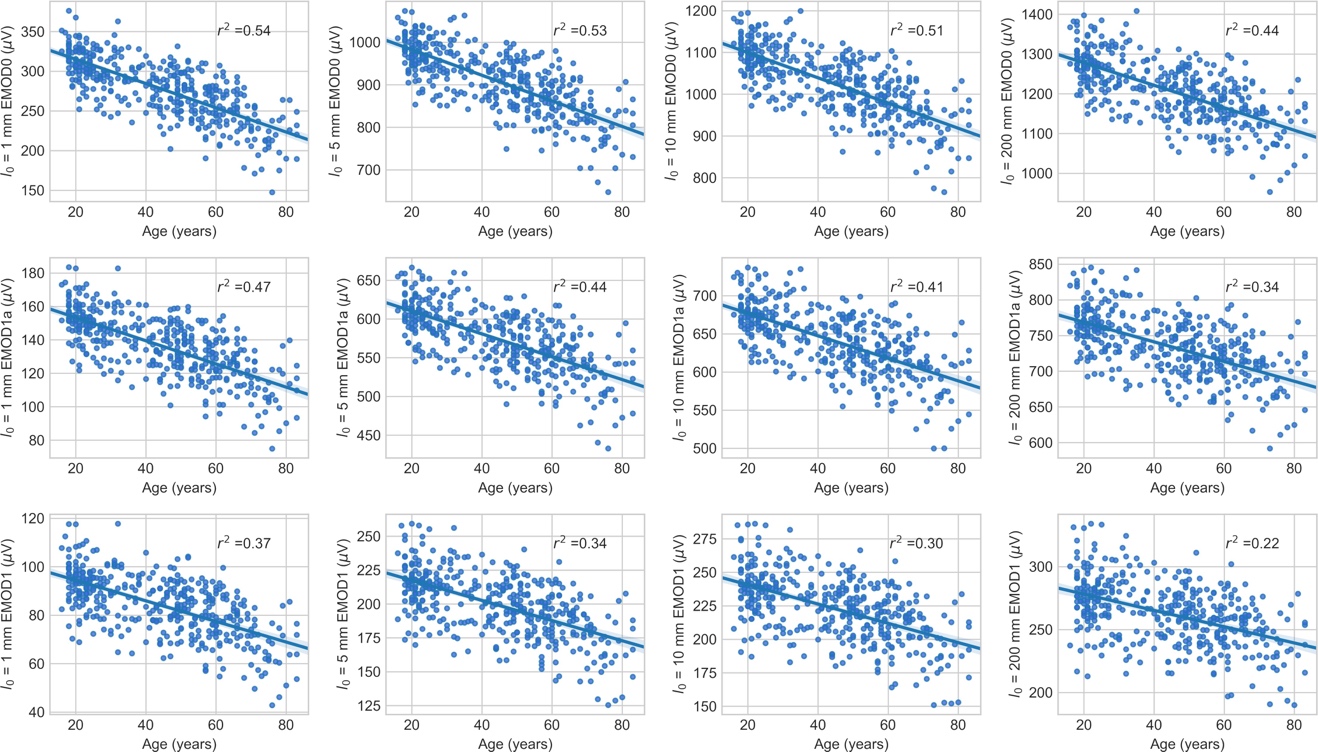

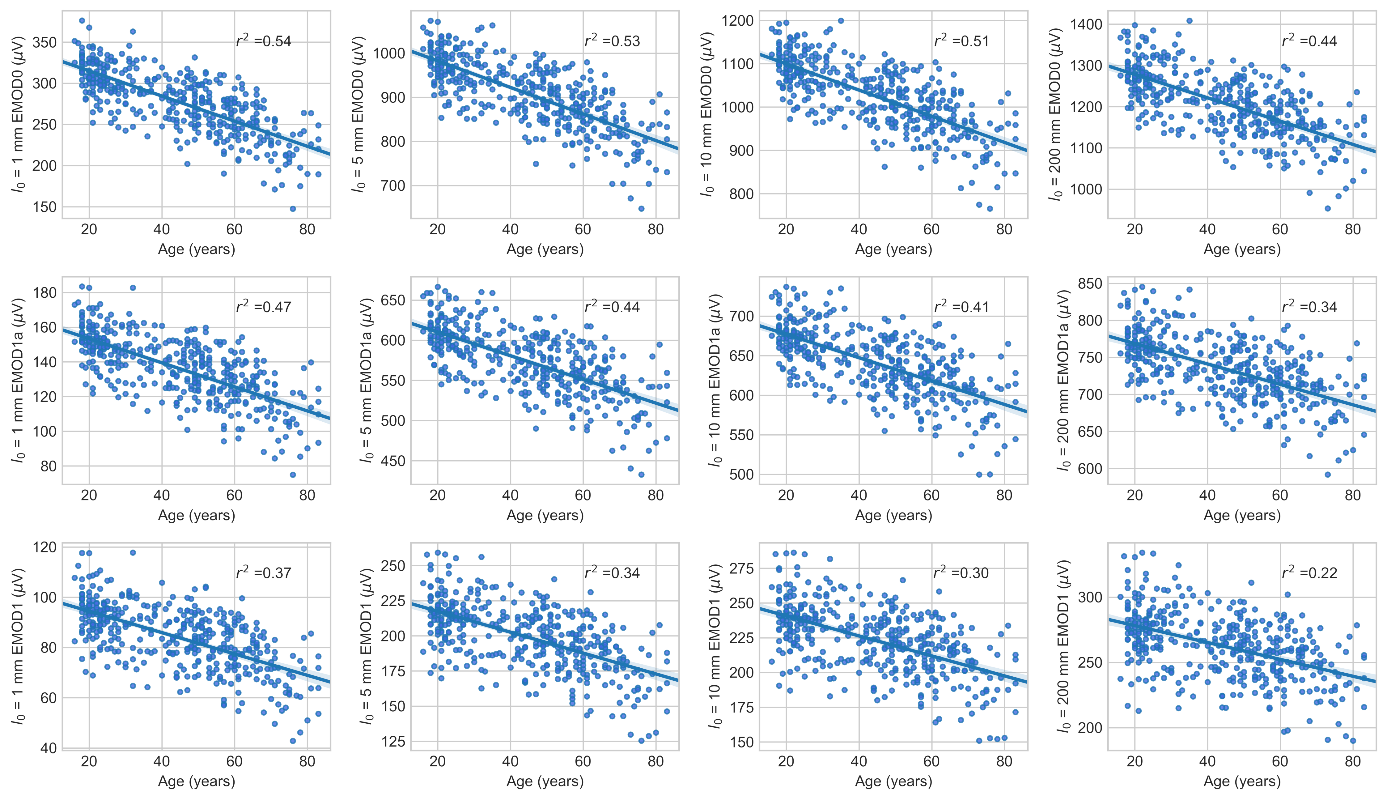


**S4 Figure:** **Linear fits of EMOD variants to age.** Different rows correspond to different EMOD1 variants: EMOD0 ($\varepsilon_{0}^{g}$), EMOD1a ($\varepsilon_{1a}^{g}$) and EMOD1 ($\varepsilon_{1}^{g}$). Different columns correspond to different l0 parameters: 1, 5, 10 and 200 mm, respectively from left to right.


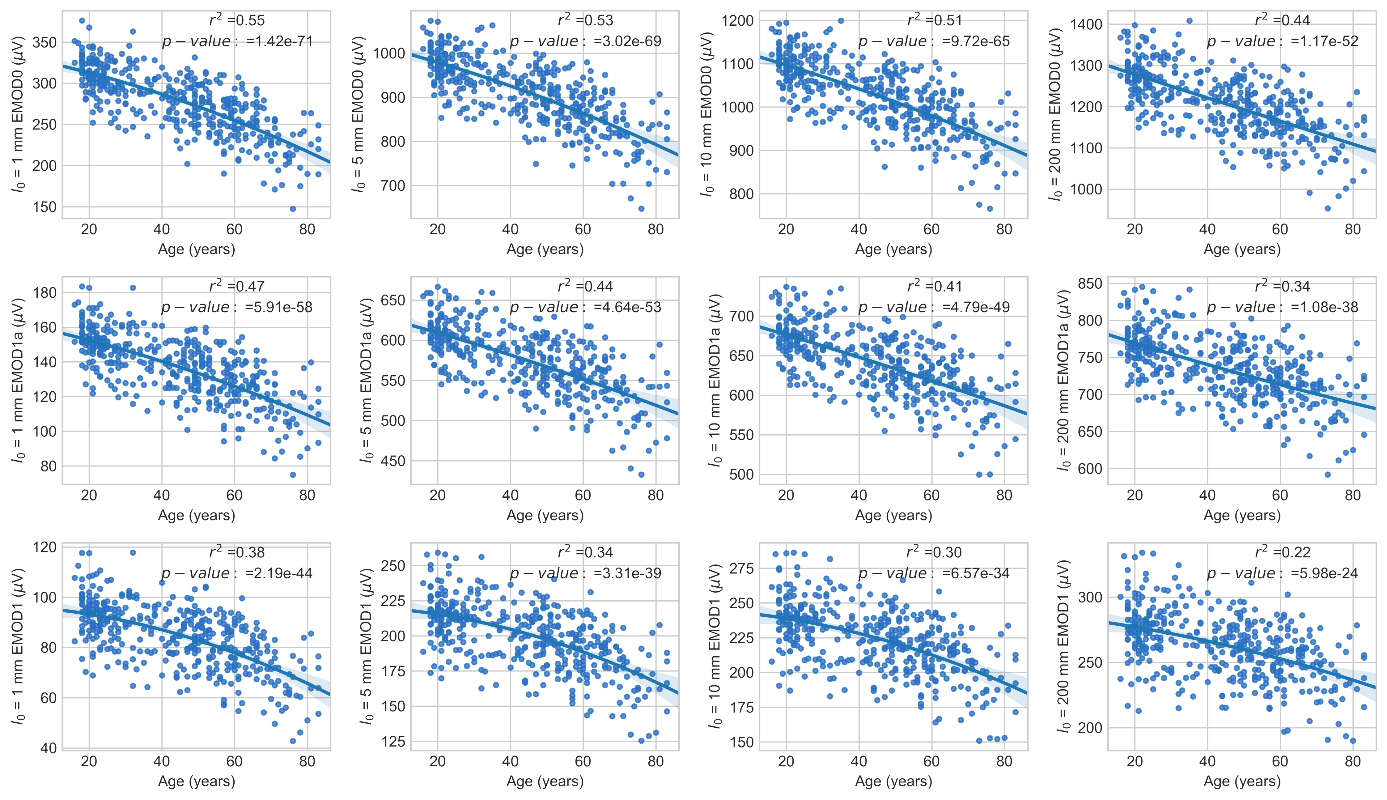


**S5 Figure**: **Second order fits of EMOD variants to age.** Different rows correspond to different EMOD1 variants: EMOD0 ($\varepsilon_{0}^{g}$), EMOD1a ($\varepsilon_{1a}^{g}$) and EMOD1 ($\varepsilon_{1}^{g}$). Different columns correspond to different *l*_0_ parameters: 1, 5, 10 and 200 mm, respectively from left to right.


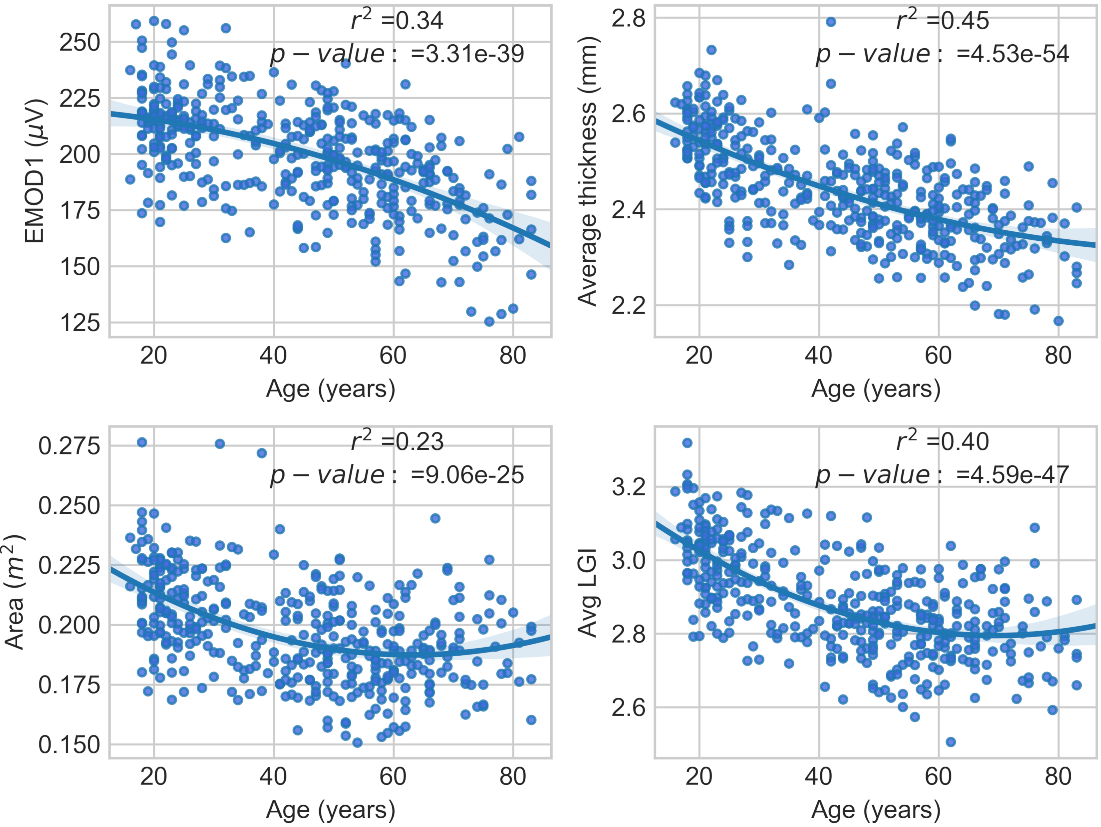


**S6 Figure:** **Second order fits of EMOD1, average LGI, average cortical thickness and cortical area to age.** For each plot, r-squared and p-values for the fit are shown as well.


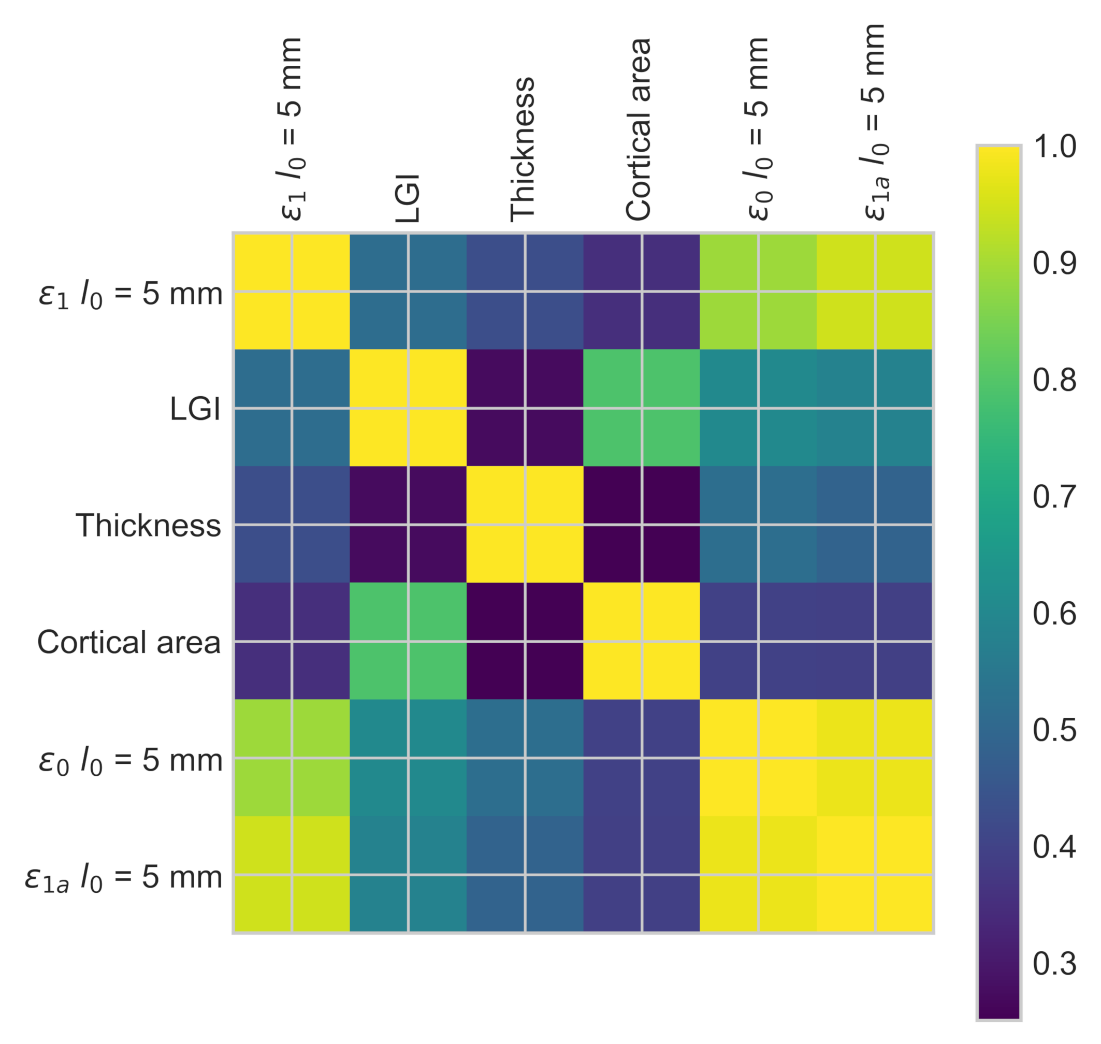


**S7 Figure:** **Pearson correlation coefficients** between different EMOD variants, average LGI, average cortical thickness and total surface area.


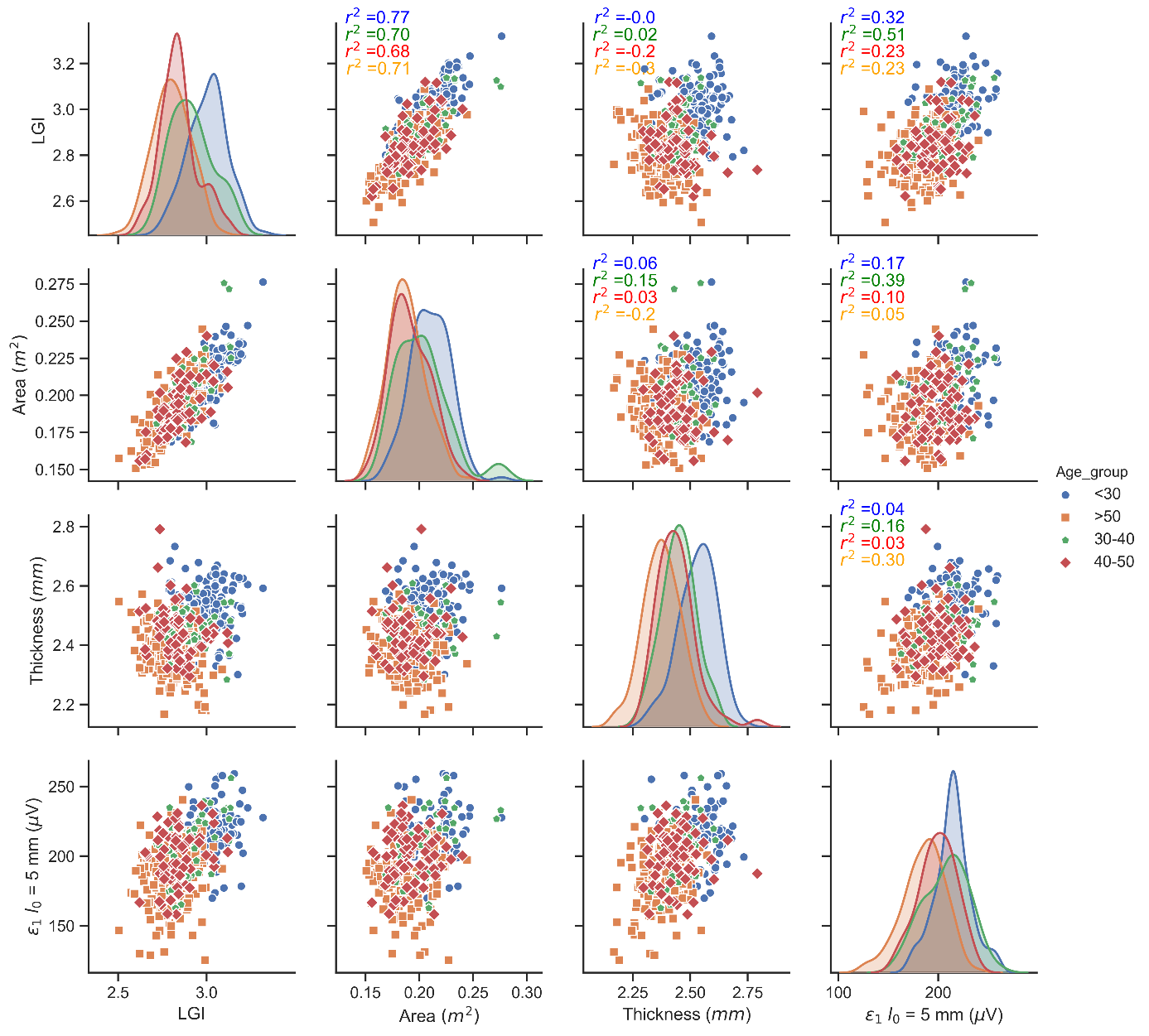


**S8 Figure**: **Correlation between average LGI, EMOD1 (***l*_0_ **set to 5 mm), average cortical thickness and total cortical area for different age range groups.** The plots along the main diagonal show histograms of these quantities grouped by age range. The offline range elements show each variable plotted against all others. Pearson correlation coefficients for each pairing, divided by age group, are also presented.


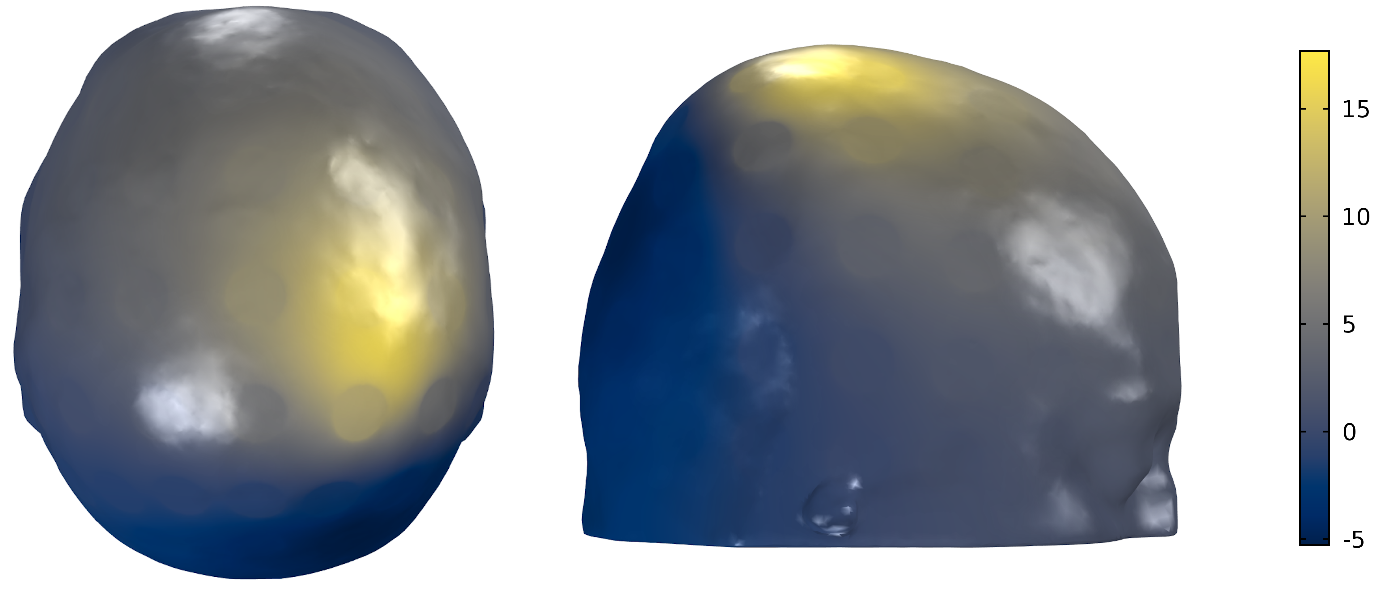


**S9 Figure**: **EEG (referenced to T8, in µV) as generated by cortical patch in Fig 3 (see also Table 1).** The dipole patch consists of 133 dipole sources (patch area of 5.3 cm^2^), with a dipole density of 0.5 nAm/mm^2^.


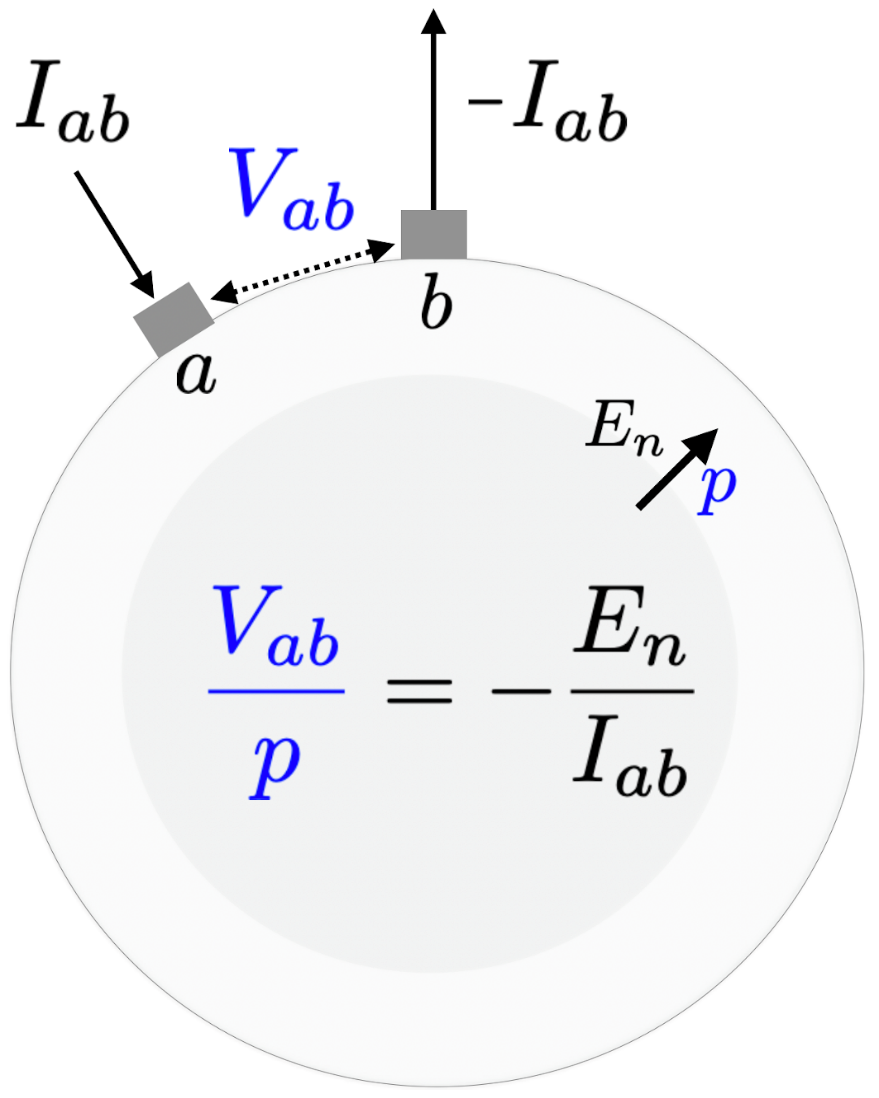


**S10 Figure**: **Illustration of reciprocity theorem for a bipolar montage**. Consider a hypothetical reciprocal EEG measurement where we observe a potential difference $V_{ab}$ between the same points $a$ and $b$ produced by a dipole *p* located at *x* and normal to the cortical surface. The reciprocity theorem implies that we can replace the pair ($E_{n},I_{ab}$) with ($V_{ab},p$) with the ratio of the first pair the same as the ratio of the second. Hence, from the current-electric field data pair we can deduce, given $V_{ab}$, a value for a reciprocal dipole $p$: $V_{ab}/p = -E_{n}/I_{ab}$.
