# Supplementary figures and images for: Realistic modeling of mesoscopic ephaptic coupling in the human brain
Source: PLoS Comput Biol. 2020 Jun 1;16(6):e1007923. doi: 10.1371/journal.pcbi.1007923 (PMC7289436; doi:10.1371/journal.pcbi.1007923)

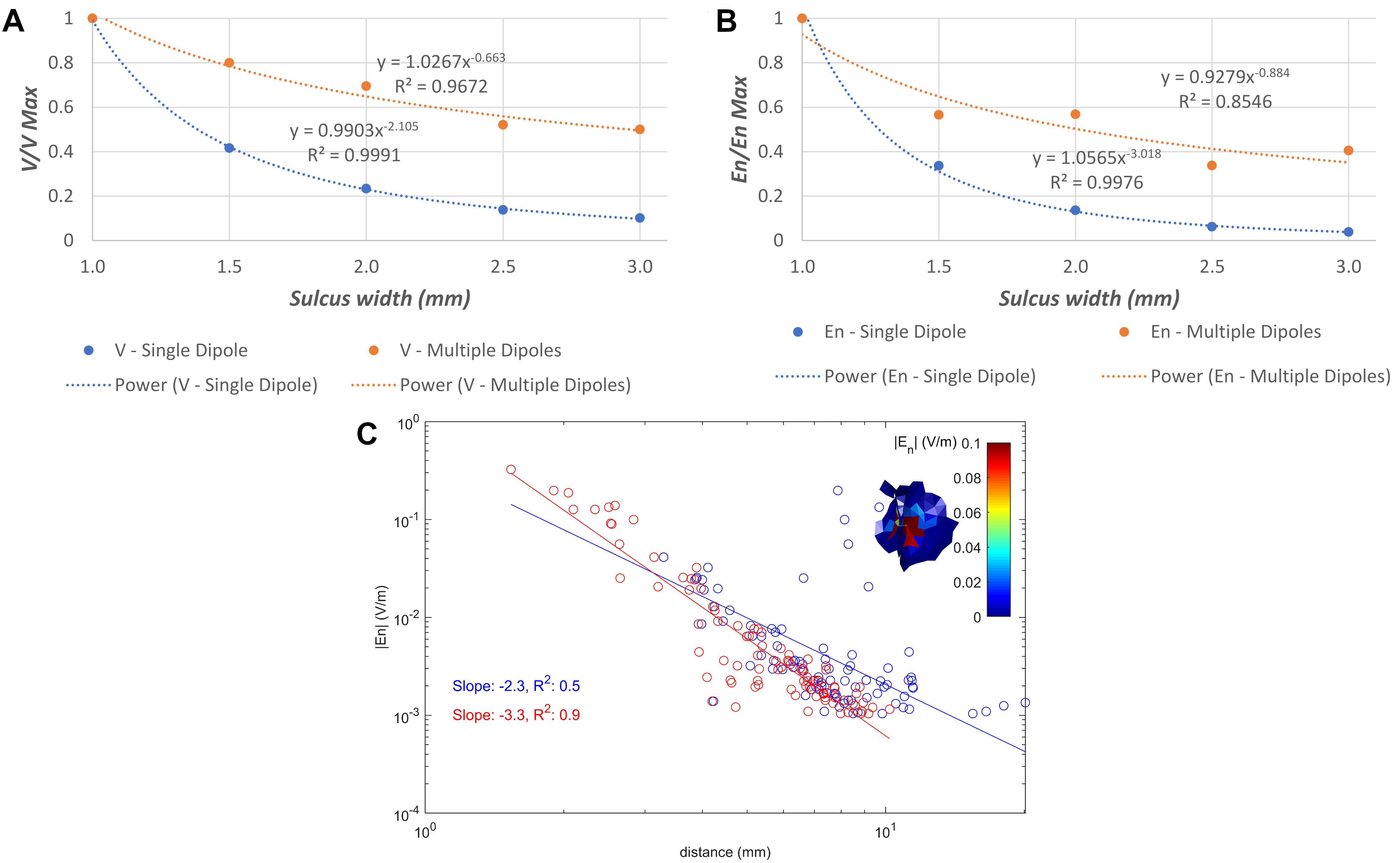

Supplement: S1 Fig — (A) Decay of V with sulcus width in the single source model (blue dots) and multiple sources model (orange dots). The fit to a power function is also shown for each model. (B) Same as (A), but now for En, the component of the electric field normal to the sulcus wall. (C) Field decay in 3D model: loglog plot of |En| (in V/m) in the GM-CSF surface as a function of the logarithm of the geodesic (blue dots) or Euclidean (red dots) distance (in mm) to the dipole. The inset shows En (in V/m) in a 3D rendering of the cortical surface. The location of the source is indicated by the red arrow. Only points where the absolute value of En is between 0.001 V/m and 1.0 V/m are shown. Linear fits to these plots are also shown, together with the slope and R2 values. (TIF) [file pcbi.1007923.s002.tif]

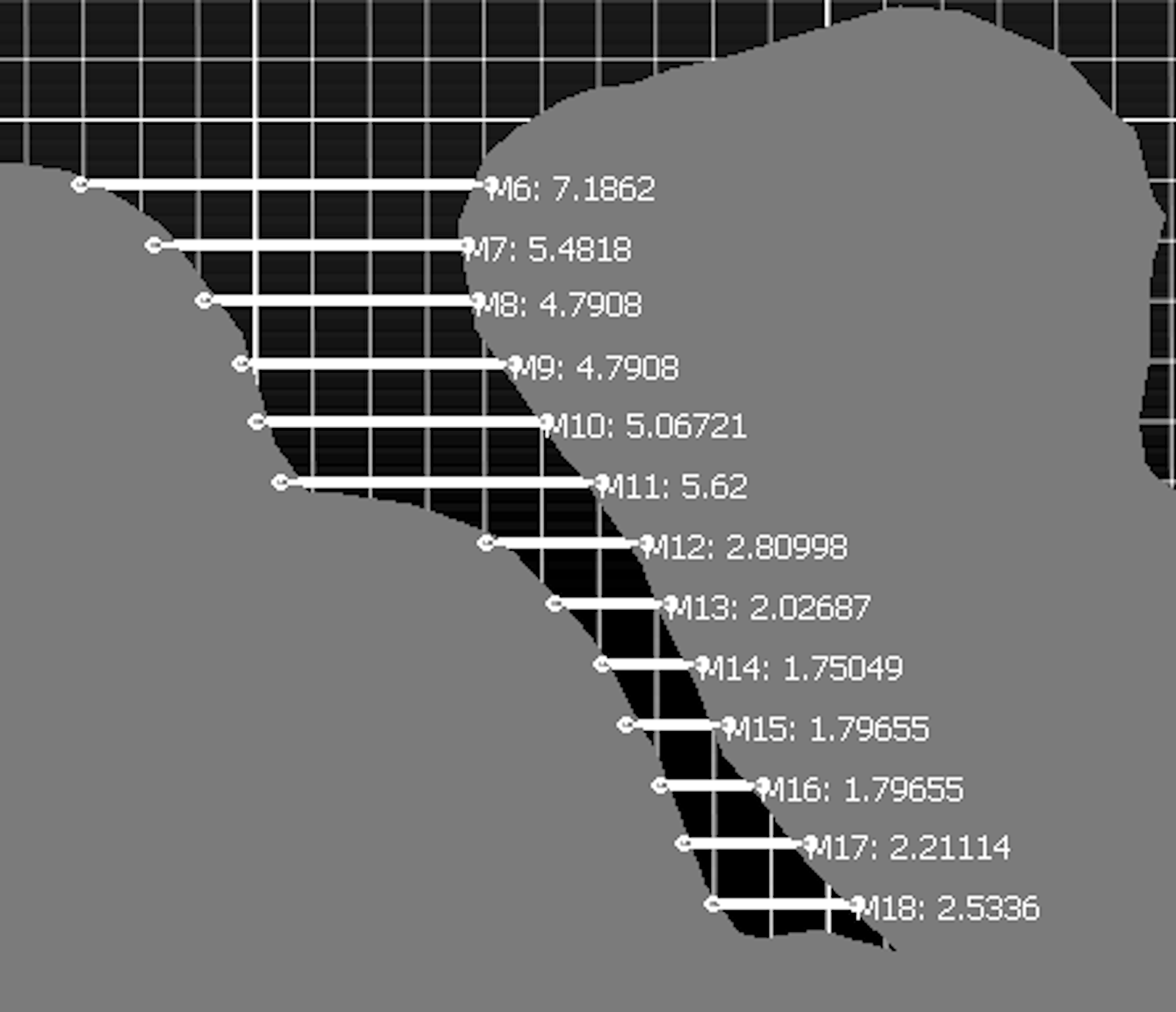

Supplement: S2 Fig — Measurements of width (mm) in the sulcus used for realistic modeling in Fig 3 in the main text. Note that this is an easy to compute approximation (bounded from above) to the minimal distance between sulcal wall points. (TIF) [file pcbi.1007923.s003.tif]

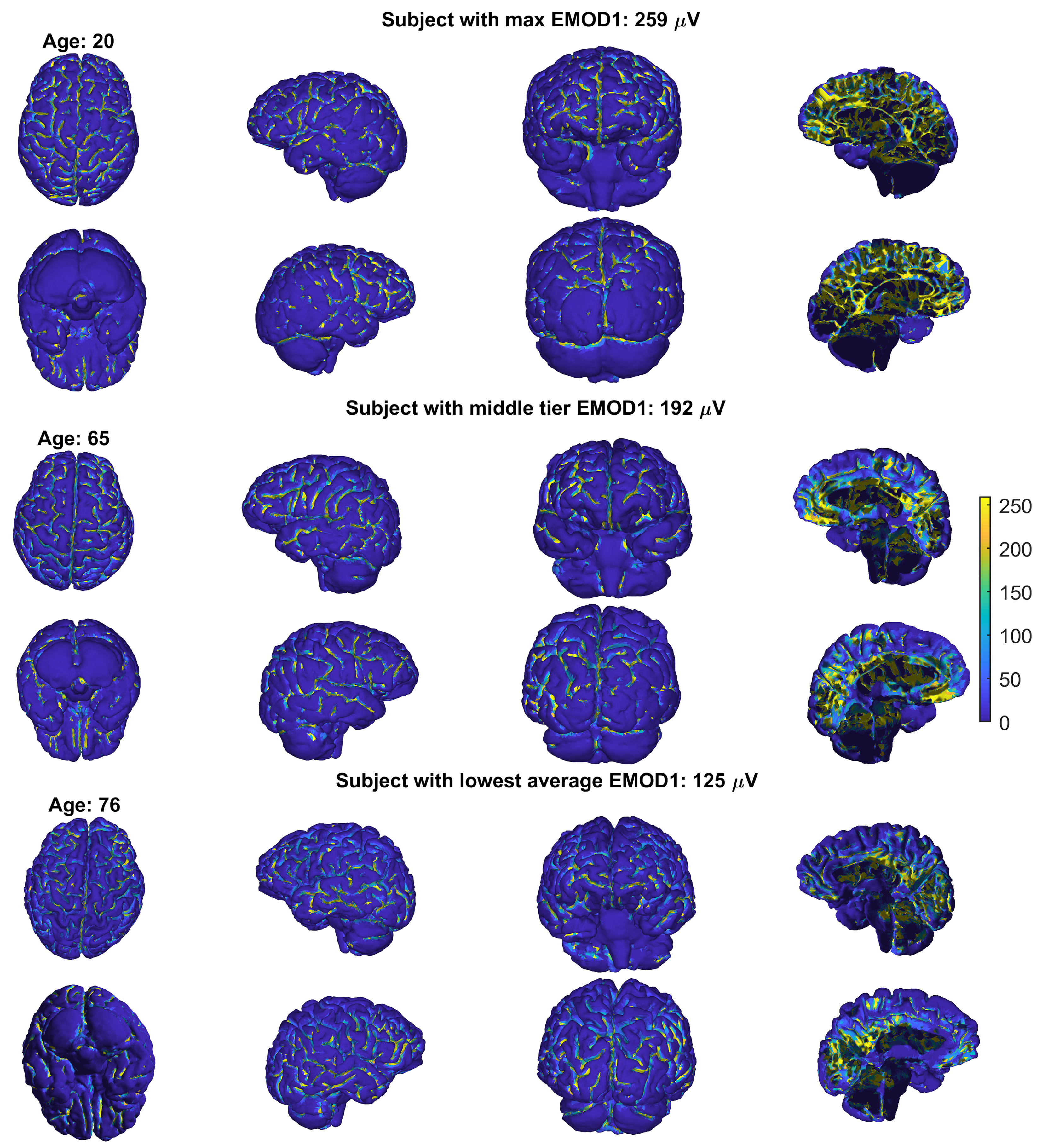

Supplement: S3 Fig — Subjects are presented from highest (top) to lowest EMOD1 (bottom) values. The color scale is common across all the plots. From left-right: top/bottom view, left/right-hemisphere view, front/back view, mid sagittal place left/right hemisphere view. (TIF) [file pcbi.1007923.s004.tif]

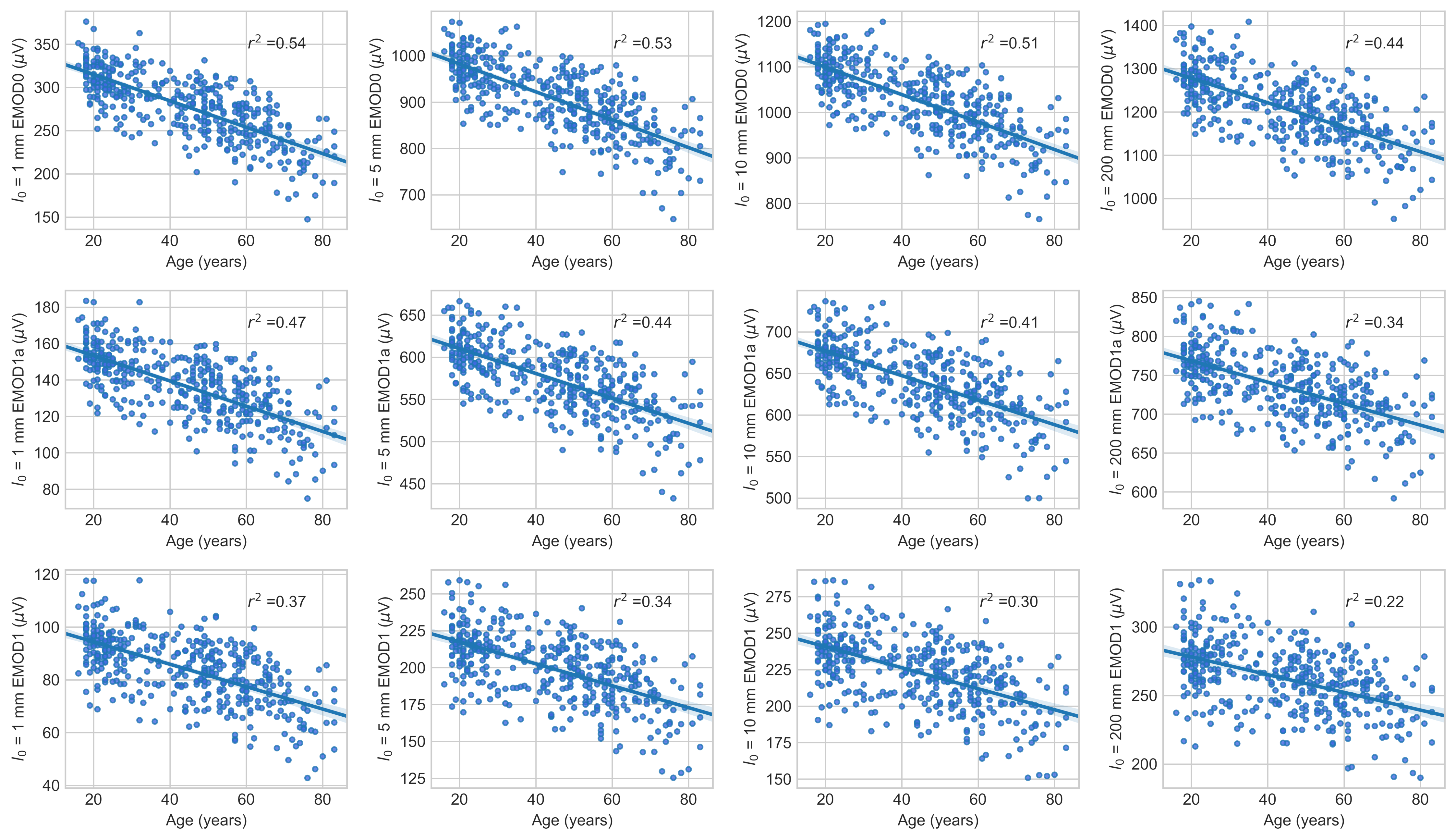

Supplement: S4 Fig — Different rows correspond to different EMOD1 variants: EMOD0 (ε0g), EMOD1a (ε1ag) and EMOD1 (ε1g). Different columns correspond to different l0 parameters: 1, 5, 10 and 200 mm, respectively from left to right. (TIF) [file pcbi.1007923.s005.tif]

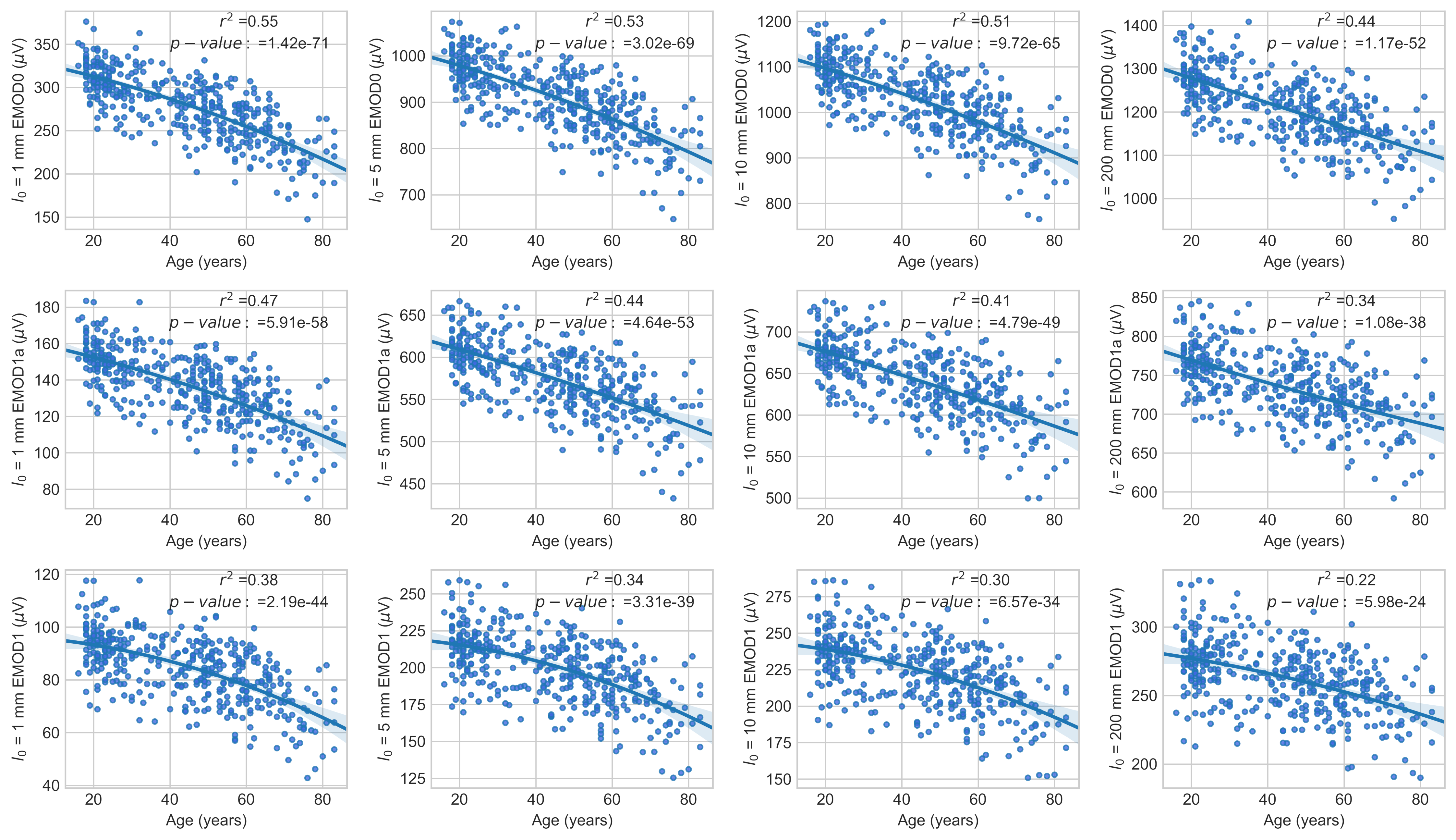

Supplement: S5 Fig — Different rows correspond to different EMOD1 variants: EMOD0 (ε0g), EMOD1a (ε1ag) and EMOD1 (ε1g). Different columns correspond to different l0 parameters: 1, 5, 10 and 200 mm, respectively from left to right. (TIF) [file pcbi.1007923.s006.tif]

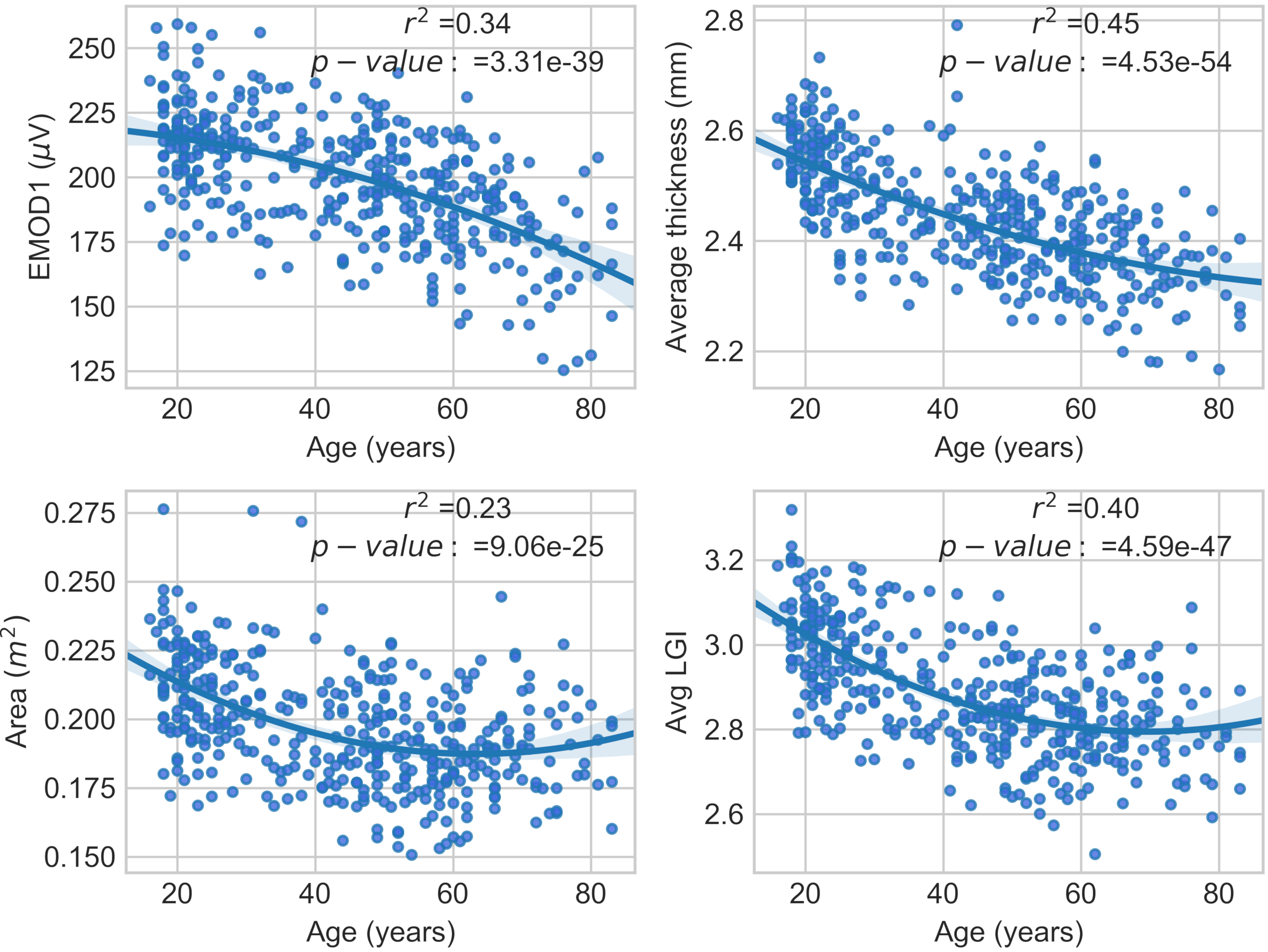

Supplement: S6 Fig — For each plot, r-squared and p-values for the fit are shown as well. (TIF) [file pcbi.1007923.s007.tif]

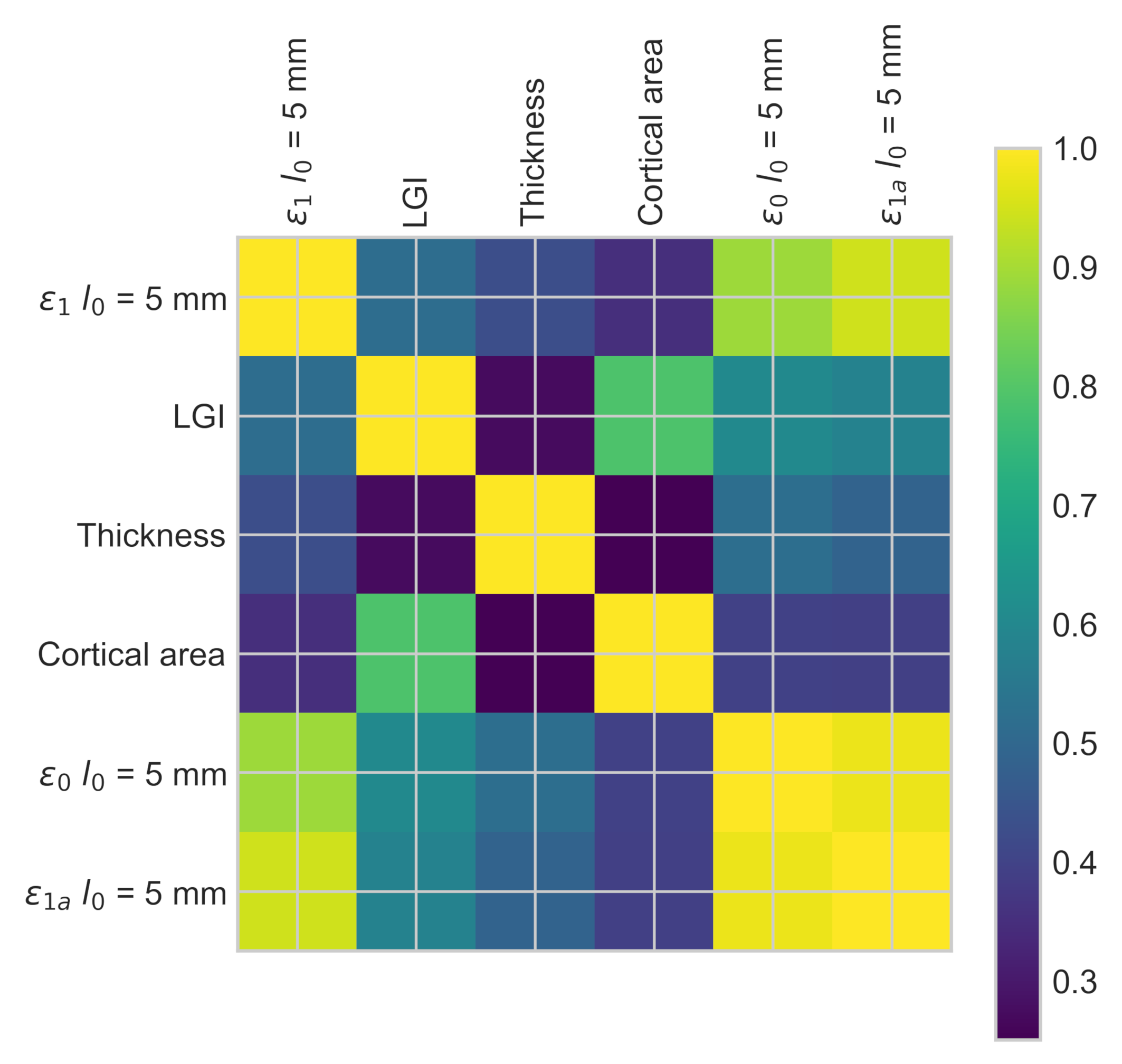

Supplement: S7 Fig — (TIF) [file pcbi.1007923.s008.tif]

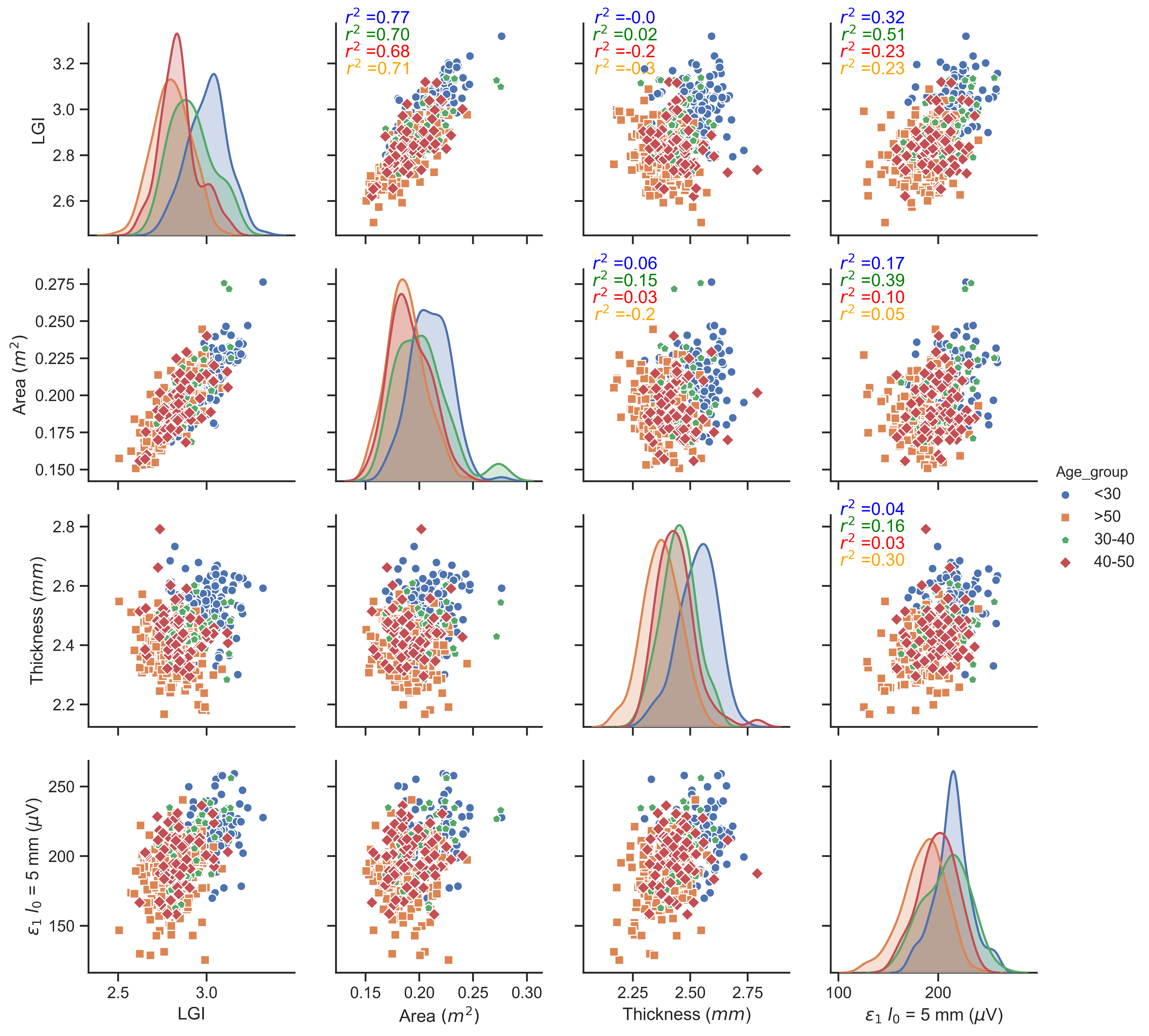

Supplement: S8 Fig — The plots along the main diagonal show histograms of these quantities grouped by age range. The offline range elements show each variable plotted against all others. Pearson correlation coefficients for each pairing, divided by age group, are also presented. (TIF) [file pcbi.1007923.s009.tif]

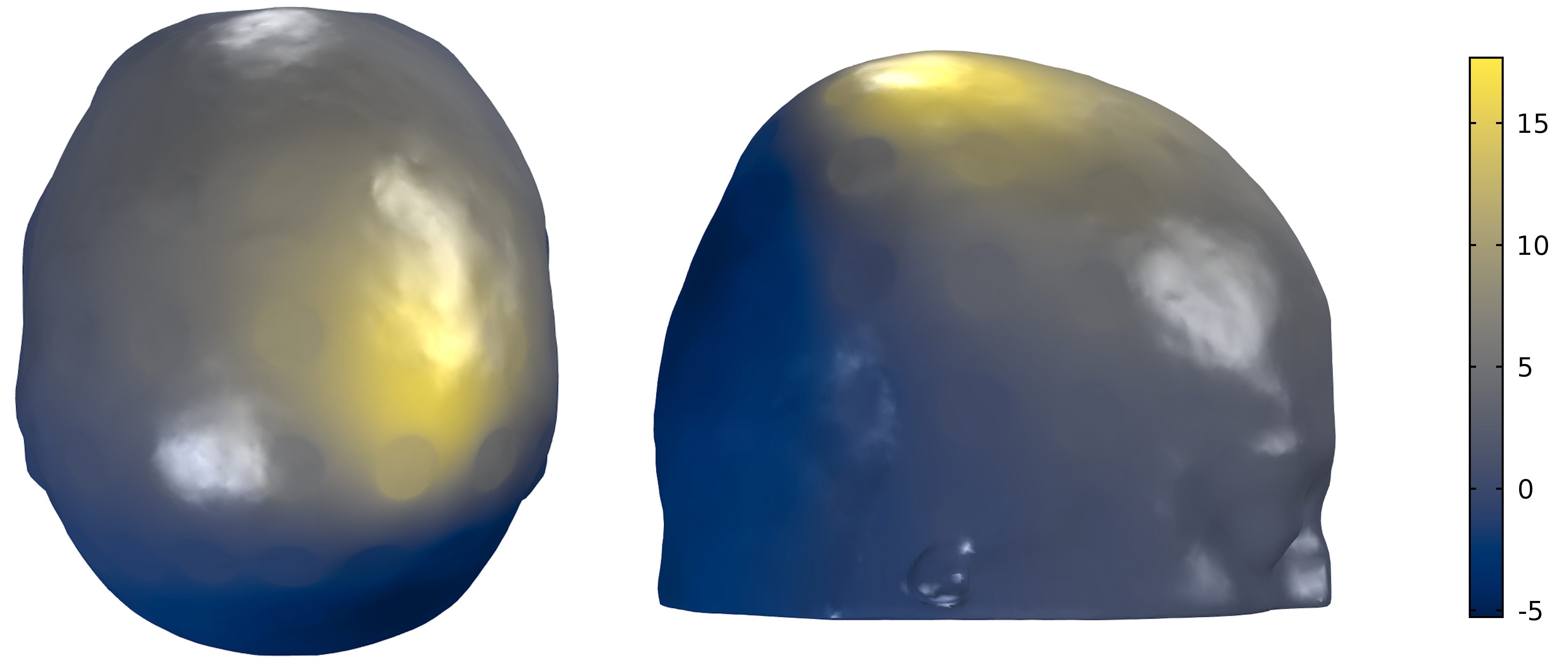

Supplement: S9 Fig — The dipole patch consists of 133 dipole sources (patch area of 5.3 cm2), with a dipole density of 0.5 nAm/mm2. (TIF) [file pcbi.1007923.s010.tif]

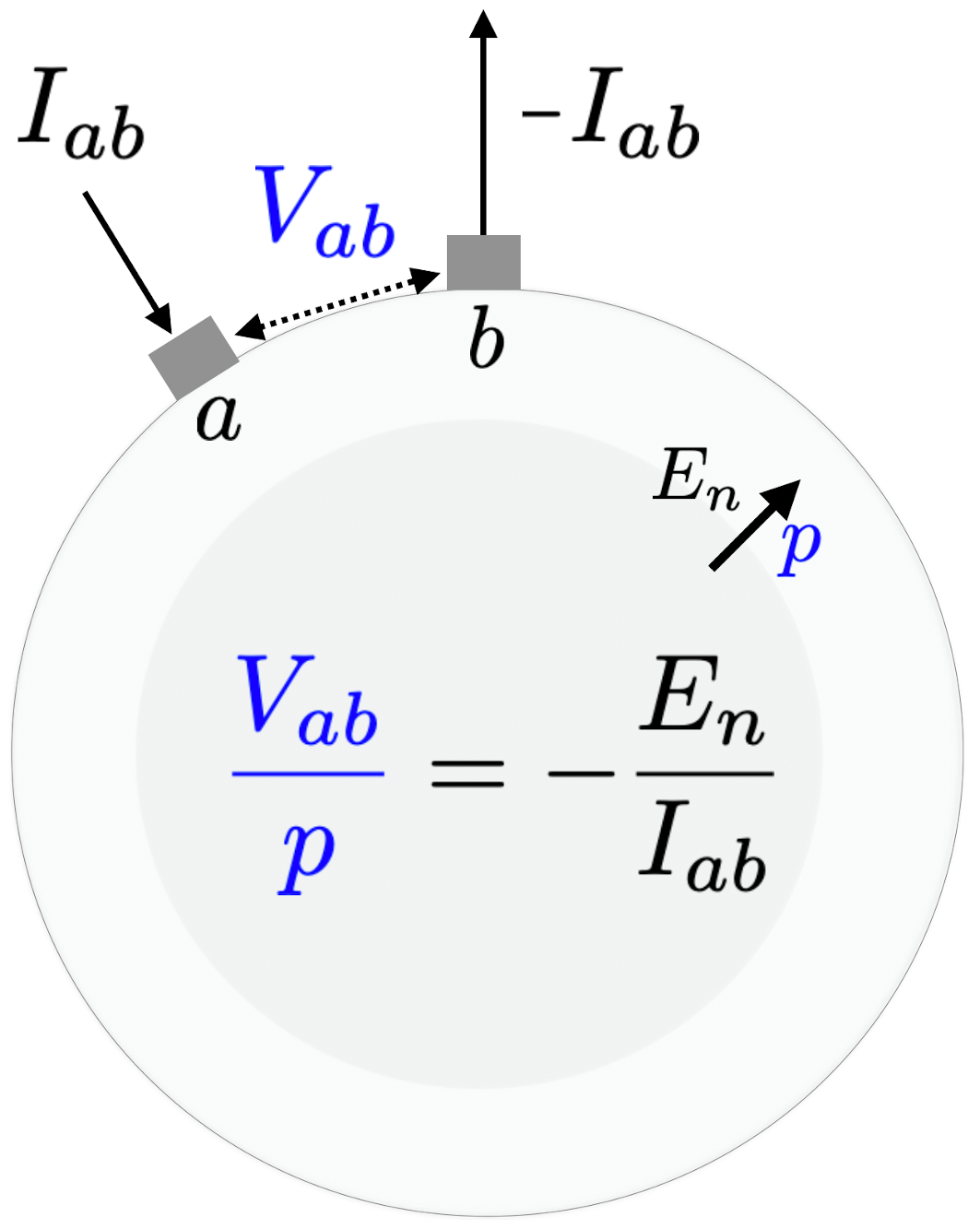

Supplement: S10 Fig — Consider a hypothetical reciprocal EEG measurement where we observe a potential difference Vab between the same points a and b produced by a dipole p located at x and normal to the cortical surface. The reciprocity theorem implies that we can replace the pair (En,Iab) with (Vab,p) with the ratio of the first pair the same as the ratio of the second. Hence, from the current-electric field data pair we can deduce, given Vab, a value for a reciprocal dipole p: Vab/p = −En/Iab. (TIF) [file pcbi.1007923.s011.tif]
